# Towards a general ruthenium-catalyzed hydrogenation of secondary and tertiary amides to amines†
†Electronic supplementary information (ESI) available: General procedures, additional tables and schemes, characterisation data and NMR spectra of the isolated compounds are available. See DOI: 10.1039/c5sc04671h


**DOI:** 10.1039/c5sc04671h

**Published:** 2016-02-09

**Authors:** Jose R. Cabrero-Antonino, Elisabetta Alberico, Kathrin Junge, Henrik Junge, Matthias Beller

**Affiliations:** a Leibniz-Institut für Katalyse e.V. , Albert Einstein Str. 29a , 18059 Rostock , Germany . Email: matthias.beller@catalysis.de; b Istituto di Chimica Biomolecolare , Consiglio Nazionale delle Ricerche , Tr. La Crucca 3 , 07100 Sassari , Italy

## Abstract

A broad range of secondary and tertiary amides has been hydrogenated to the corresponding amines under mild conditions using an *in situ* catalyst generated by combining [Ru(acac)_3_], 1,1,1-tris(diphenylphosphinomethyl)ethane (Triphos) and Yb(OTf)_3_. The presence of the metal triflate allows to mitigate reaction conditions compared to previous reports thus improving yields and selectivities in the desired amines. The excellent isolated yields of two scale-up experiments corroborate the feasibility of the reaction protocol. Control experiments indicate that, after the initial reduction of the amide carbonyl group, the reaction proceeds through the reductive amination of the alcohol with the amine arising from collapse of the intermediate hemiaminal.

## Abstract 2

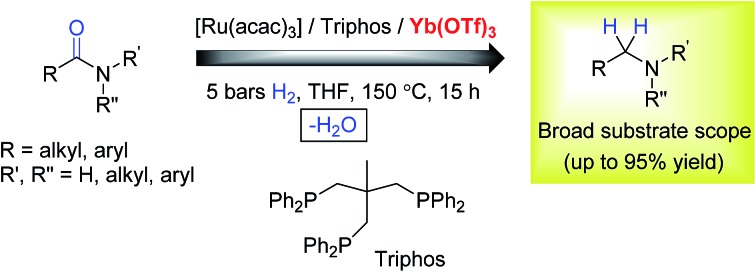
[Ru(acac)_3_]/Triphos in combination with Yb(OTf)_3_ constitutes an improved catalyst system for the hydrogenation of aliphatic and aromatic secondary and tertiary amides.

## Introduction

Amines constitute an important class of compounds which have wide industrial applications as solvents, additives, anti-foam agents, corrosion inhibitors, detergents, dyes, bactericides.^1^ In addition, the amino group is abundantly present in agrochemicals and pharmaceuticals.^1^ Although aliphatic amines can be prepared by numerous methodologies, reductive aminations including alcohol aminations prevail in industry on larger scale. In addition, primary aromatic and benzylic amines are most easily accessible by reduction of the corresponding nitroarenes and nitriles. For the preparation of more structurally complex bio-active compounds and natural products a preferred route for C–N bond formation combines amidation followed by reduction.^2^ This latter reaction is usually accomplished by using (over)stoichiometric amounts of lithium aluminum hydride (LiAlH_4_) or borane (B_2_H_6_). Unfortunately, these reagents require complex and hazardous work-up procedures and generate stoichiometric amounts of difficult to dispose waste by-products. Therefore more efficient alternatives are highly sought after. Recently, metal-promoted catalytic hydrosilylation of amides were intensely investigated and mild operational conditions have been devised which allow for excellent functional group tolerance, yet the method suffers from low atom efficiency because of residual siloxanes.^3^ Another recent example of a magnesium-catalyzed deoxygenation of amides *via* hydroboration has similar limitations.^4^ Obviously, the best option for amide reduction in terms of atom economy and waste prevention is hydrogenation with molecular hydrogen in the presence of a suitable catalyst.^5^ However, because of the low electrophilicity of their carbonyl group, amides require elevated pressures and temperatures to be reduced. In the field of heterogeneous catalysis, copper–chromium oxide catalysts originally developed to this aim have been replaced by less toxic, more efficient bifunctional/bimetallic Ru/Mo,6*a* Rh/Mo,6a,b Ru/Re,6a,c and Rh/Re catalysts6a,c and more recently by bimetallic graphite-supported Pd–Re6*d* and TiO_2_-supported Pt–Re based catalysts.6e,f Improvement of heterogeneous amide hydrogenation catalysts has witnessed a mitigation of reaction conditions, yet they are incompatible with aromatic groups and multiple CC and CX bonds which are likewise reduced. Despite significant interest in developing homogeneous catalysts which should operate under milder reaction conditions, no such general methodology is available. Interestingly, depending on the type of homogeneous catalyst, the hydrogenation of amides affords either the alcohol and amine, arising from cleavage of the C–N bond in the intermediate hemiaminal, or the more desired higher amine, resulting from deoxygenation of the amide. The former type of selectivity is preferentially achieved with bifunctional catalysts, which rely on metal–ligand cooperation, and can be advantageously exploited as a mild deprotection methodology.^7^

**Scheme 1:**
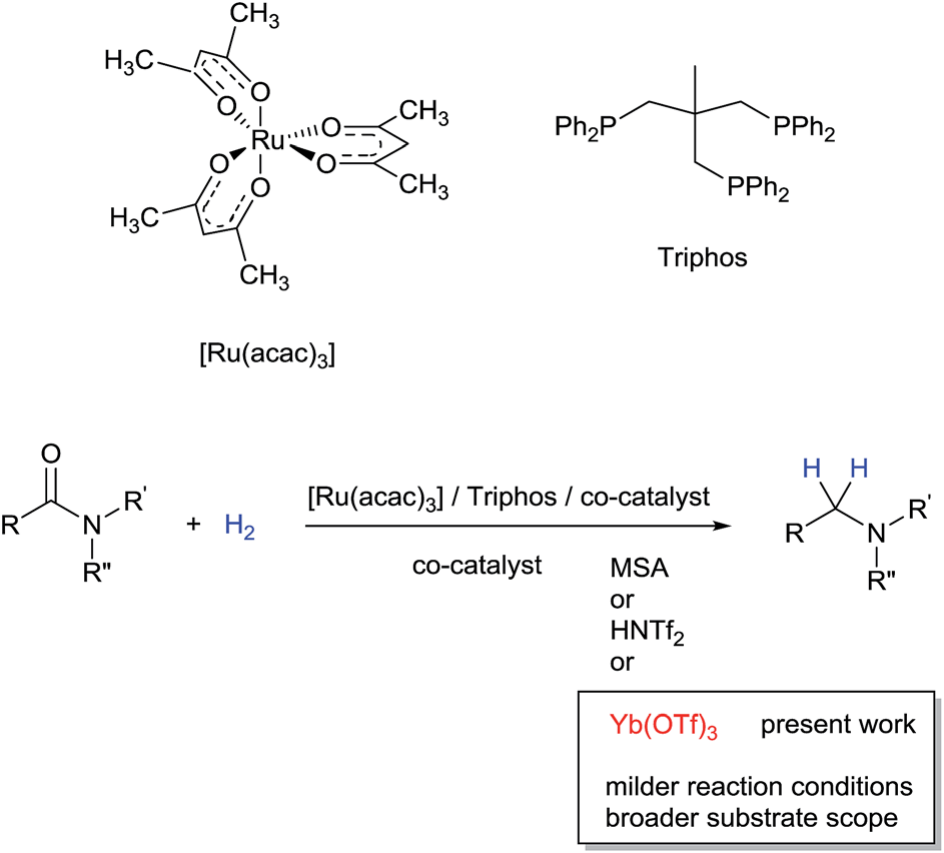
Ru/Triphos catalytic systems competent for the homogeneous hydrogenation of amides to amines.

Investigations by Cole-Hamilton and co-workers, later supported by contributions from Leitner's and Klankermayer's groups, have shown that ruthenium catalysts modified by 1,1,1-tris(diphenylphosphinomethyl)ethane (Triphos) give access to the higher amine (Scheme 1).^8^ Either the catalytic system generated *in situ* from [Ru(acac)_3_] and Triphos8a,b or the readily accessible molecularly defined complex [(Triphos)Ru(TMM)] (TMM = trimethylenemethane)8*c* were used for this transformation at high temperature. Notably, both systems require the presence of an acid co-catalyst such as methanesulfonic acid (MSA)8a–c or bis(trifluoromethane)sulfonimide (HNTf_2_)8*d* and the catalyst performance is strongly dependent on the acid/ruthenium precursor ratio. Although the individual intermediates around the catalytic cycle have so far escaped detection, an in-depth study of the ruthenium species present in solution under catalytic conditions has allowed to shed light on the role of the acid co-catalyst: it serves to convert the catalyst precursor into an active system and it provides a weakly coordinating counter anion (CF_3_S(O)_2_O^–^ or NTf_2_^–^) which, while stabilizing the [Ru(Triphos)]^2+^ fragment, does not prevent coordination of the incoming hydrogen molecule and substrate. Furthermore, it provides the optimal reaction medium p*K*_a_, to promote the H-transfer and hydrolytic events which make up amide hydrogenation.8*c*

The Ru–Triphos catalyst system is noteworthy in that, unlike heterogeneous catalysts, it does not promote the hydrogenation of aromatic moieties and, to the best of our knowledge, represents the only homogeneous catalytic system able to hydrogenate amides to afford the alkylated amine resulting from the hydrogenation of the carbonyl group and the formal hydrogenolysis of the ensuing C–OH bond. However, harsh conditions are still required to achieve this transformation and the substrate scope is limited as the catalyst is best suited for 1° amides and substrates which bear a phenyl ring directly attached to the nitrogen atom.^8^

Recently, we have shown that it is possible to extend the range of aromatic and aliphatic carboxylic acids which can be hydrogenated to the corresponding alcohols with the Ru–Triphos system by replacing the Brönsted acid co-catalyst8d,^9^ with either Sn(OTf)_2_ or Al(OTf)_3_, affording a milder and more general method for the reduction of these challenging substrates.10*a* The Lewis acid is also key to the selective hydrogenation of esters to ethers promoted by the Ru–Triphos system: while no reaction occurs with methanesulfonic acid as co-catalyst, and poor selectivity is provided by trifluoromethanesulfonic acid, the combined use of Ru–Triphos and Al(OTf)_3_ gives access to cyclic and linear ethers in good to excellent yields.10*b*

Inspired by these results, we envisaged that the presence of a suitable Lewis acid might be crucial for the reduction of amides as well, by activating the carbonyl group and thus allowing mitigation of the reaction conditions. Herein, we present for the first time the results of our detailed investigations which have led us to identify a superior catalyst system for the selective hydrogenation of a broad range of amides to amines. Moreover, mechanistic investigations revealed a novel pathway for the hydrogenation of amides.

## Results and discussion

In order to assess whether a Lewis acid could indeed be a beneficial co-catalyst for the transformation under investigation, preliminary experiments were performed using the hydrogenation of benzanilide **1** as benchmark reaction and the metal triflates readily available in the laboratory. The reactions were run using the catalytic system generated *in situ* from [Ru(acac)_3_] and Triphos in THF at 150 °C under 50 bar of H_2_ for a standard reaction time of 15 hours in the presence of 1 equivalent of the Lewis acid as to ruthenium. The first co-catalysts to be tested were Sn(OTf)_2_ and Al(OTf)_3_ as these had proved effective in co-catalyzing the reduction of carboxylic acids10*a* and the selective hydrogenation of esters to ethers respectively.10*b* While Sn(OTf)_2_ gave poor conversion with no selectivity in the desired *N*-benzylaniline **2**, the only products being benzyl alcohol **3** and aniline **4** (Table 1, entry 1),^11^ Al(OTf)_3_ afforded full conversion, although with a modest 27% selectivity (Table 1, entry 2). Other three metal triflates were investigated: In(OTf)_3_, Sc(OTf)_3_, Hf(OTf)_4_ (Table 1, entries 3, 4, 5). Essentially quantitative conversions were achieved in all cases, with Hf(OTf)_4_ providing the highest yield, 34%, of *N*-benzylaniline **2**. The use of a 2-fold excess of the same Lewis acid as to Ru allowed an increase in the yield of product **2** (42%, Table 1, entry 6) but larger amounts were detrimental (Table 1, entry 7 and 8).

**Table 1:** Hydrogenation of benzanilide **1** with the Ru/Triphos catalyst: preliminary explorative experiments into suitable additives

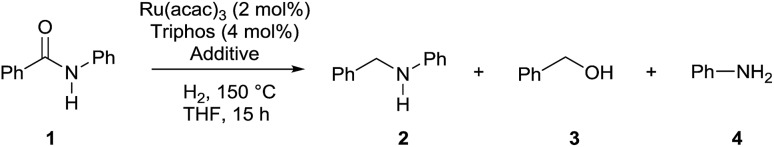					
Entry^*a*^	Additive (mol%)	Conv.^*b*^ (%)	**2** ^*b*^ (%)	**3** ^*b*^ (%)	**4** ^*b*^ (%)
1	Sn(OTf)_2_ (2)	25	—	25	25
2	Al(OTf)_3_ (2)	>99	27	71	71
3	In(OTf)_3_ (2)	93	23	68	67
4	Sc(OTf)_3_ (2)	>99	29	67	66
5	Hf(OTf)_4_ (2)	>99	34	63	60
6	Hf(OTf)_4_ (4)	>99	42	51	37
7	Hf(OTf)_4_ (6)	>99	40	54	39
8	Hf(OTf)_4_ (10)	73	17	32	20
9^*c*^	Hf(OTf)_4_ (4)	>99	63	32	19
10^*c*^	HOTf (16)	84	41	39	26
11^*c*^	—	5	—	3	3

^*a*^Standard reaction conditions: benzanilide **1** (100.6 mg, 0.5 mmol), Ru(acac)_3_ (2 mol%), Triphos (4 mol%), additive (2–16 mol%), THF (2 mL) and H_2_ (50 bar) at 150 °C, reaction time 15 h.

^*b*^Conversion of **1** and yields of **2**, **3**, and **4** were calculated by GC using hexadecane as internal standard. In some cases, variable amounts of *N*-phenylpyrrolidine (5–15%) were produced following acid promoted ring-opening of THF.

^*c*^Reactions were run under 15 bar H_2_.

Having established the optimal Hf(OTf)_4_/Ru ratio, this was applied to probe the influence of temperature and pressure on selectivity: the latter increases at higher temperature and lower pressure (Table S1†), in line with previous findings.8a,c Therefore by reducing the hydrogen pressure to 15 bar while keeping the temperature at 150 °C, the yield of *N*-benzylaniline **2** rose further to 63% (Table 1, entry 9). A control experiment was run in the presence of triflic acid (HOTf), as this might arise from the hydrolysis of the corresponding metal salt (Table 1, entry 10): while a 84% conversion was achieved, thus suggesting the possibility of a background reaction promoted by the Brönsted acid, the selectivity in **2** was lower (49%) than the one obtained with Hf(OTf)_4_ (63%), establishing the positive influence of the metal ion.

Under the same experimental conditions, almost no reaction took place in the absence of the Lewis acid (Table 1, entry 11).

**Fig. 1:**
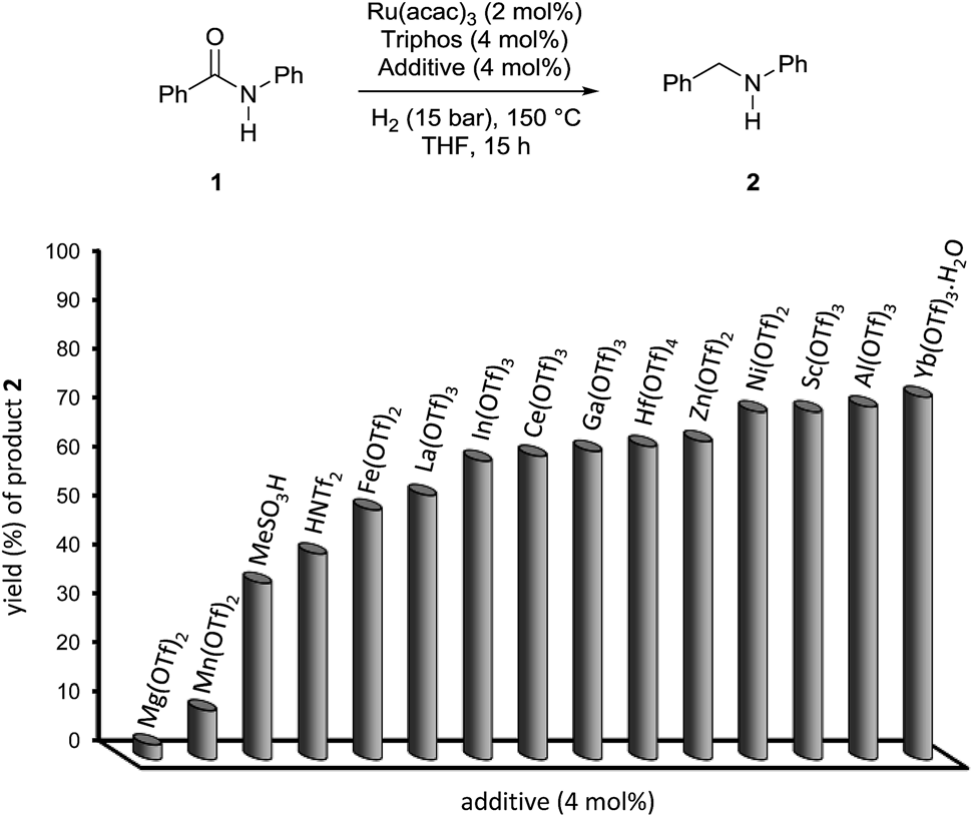
Hydrogenation of benzanilide **1** with the Ru/Triphos catalyst: yield of amine **2** in the presence of different Lewis and Brönsted acid co-catalysts. Reaction conditions: benzanilide **1** (100.6 mg, 0.5 mmol), Ru(acac)_3_ (2 mol%), Triphos (4 mol%), additive (4 mol%), THF (2 mL) and H_2_ (15 bar) at 150 °C during 15 h. Yield of **2** was calculated by GC using hexadecane as internal standard.

The results of this preliminary survey were deemed encouraging and the screening of a wider range of metal triflates worthwhile: the results are summarized in Fig. 1 and Table S2.†

Triflates of several transition metals, of the third group metals and of a few of the rare-earth ones, all promoted quantitative conversion of benzanilide **1** under the applied conditions. However, the yield in *N*-benzylaniline **2** was highly affected by the type of metal going from a minimum of 3% with Mg(OTf)_2_ up to 74% with Yb(OTf)_3_·H_2_O. Al(OTf)_3_ afforded a comparable yield, 72%, as to Yb(OTf)_3_·H_2_O. Despite the slightly lower cost of the former,^12^ Yb(OTf)_3_·H_2_O was selected as the co-catalyst of choice in light of the positive characteristics shown by rare-earth metal triflates such as increased stability towards moisture and air, compatibility with many Lewis bases containing nitrogen, oxygen, phosphorus and sulfur atoms (which might be beneficial for functional group tolerance) and the possibility of being recycled at the end of the reaction, for which they are regarded as environmentally friendly.^13^ Finally, none of the tested Brönsted acids, methanesulfonic acid (MSA), bis(trifluoromethane)sulfonimide (HNTf_2_) and the already mentioned triflic acid outperformed Yb(OTf)_3_·H_2_O as co-catalyst under the same experimental conditions.

Having identified in Yb(OTf)_3_·H_2_O the Lewis acid of choice, more detailed investigations of the reaction conditions were carried out.

Selectivity in the amine **2** turned out to be affected by the Yb(OTf)_3_·H_2_O/ruthenium ratio and either less (Table 2, entry 1) or more (Table 2, entry 3) than 2 (Table 2, entry 2) was detrimental. This ratio was therefore applied in all subsequent experiments. When the amount of catalyst was reduced (Table 2, entry 4) activity was retained as conversion was quantitative but selectivity decreased from 74% (Table 1, entry 2) to 68%. By reducing the hydrogen pressure further from 15 to 5 bar the yield of *N*-benzylaniline **2** improved from 74% (Table 2, entry 2) to 85% (Table 2, entry 5). Carrying out the reaction in the presence of molecular sieves to remove water and shift the equilibrium towards the desired amine did not affect conversion but had a dramatic impact on selectivity affording more than 90% of benzyl alcohol **3** and aniline **4** (Table 2, entry 6). Addition of a controlled amount of water, 10% v/v as to the amount of the solvent THF, almost halved conversion but afforded the same selectivity provided by anhydrous conditions (Table 2, entry 7). Therefore the adventitious water present in the solvent and that produced by the reaction itself is required for optimal performance, either none or more again is detrimental. When the reaction was run in the absence of Triphos, with (Table 2, entry 8) or without Lewis acid (Table 2, entry 9), conversion was low and products arising from hydrogenation of the aromatic rings were observed, none of which was detected under otherwise identical experimental conditions, suggesting that the reactions are indeed homogeneous. The presence of Yb(OTf)_3_·H_2_O is essential to promote the reaction under such mild conditions, as no conversion of benzamide **1** at all is observed with the sole Ru–Triphos catalyst (Table 2, entry 10). At such low pressure, replacing the Lewis acid with an amount of triflic acid equivalent to that expected from its complete hydrolysis reduces conversion from quantitative to 67% with an even more dramatic impact on selectivity in **2** which drops from 85 to 37% (Table 2, entry 11).

**Table 2:** Hydrogenation of benzanilide **1** with [Ru/Triphos/Yb(OTf)_3_·H_2_O] system: fine tuning of reaction conditions

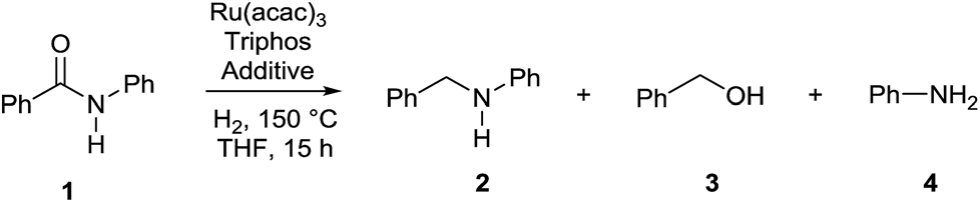							
Entry^*a*^	H_2_ (bar)	[Ru] (mol%)	[Yb] (mol%)	Conv.^*b*^ (%)	**2** ^*b*^ (%)	**3** ^*b*^ (%)	**4** ^*b*^ (%)
1	15	2	2	>99	65	35	28
2	15	2	4	>99	74	24	15
3	15	2	6	>99	72	25	13
4	15	1	4	>99	68	33	24
5	5	2	4	>99	85	14	6
6^*c*^	5	2	4	>99	5	90	94
7^*d*^	5	2	4	59	2	54	57
8^*e*^	5	2	4	44	—	—	—
9^*e*^	5	2	—	32	—	—	—
10	5	2	—	—	—	—	—
11^*f*^	5	2	—	67	25	72	75

^*a*^Standard reaction conditions: benzanilide **1** (100.6 mg, 0.5 mmol), Ru(acac)_3_ (1–2 mol%), Triphos (2 eq. respect to Ru), Yb(OTf)_3_·H_2_O (2–6 mol%), THF (2 mL) and H_2_ (5 or 15 bar) at 150 °C over 15 h. In all reactions the autoclave was purged with 30 bar of hydrogen for three times. [Ru] = [Ru(acac)_3_] and [Yb] = [Yb(OTf)_3_·H_2_O] correspond to mol% of each species.

^*b*^Conversion of **1** and yields of **2**, **3**, and **4** were calculated by GC using hexadecane as internal standard. In some cases, variable amounts of *N*-phenylpyrrolidine (5–10%) were produced following Yb(OTf)_3_·H_2_O promoted ring-opening of THF.

^*c*^Run with molecular sieves (4 Å).

^*d*^Run with 0.2 mL of water.

^*e*^Run without Triphos. The only products observed were hydrogenation ring products.

^*f*^Run in the presence of HOTf (12 mol%).

Considering the key role of the Lewis acid, the possibility that it might be as well effective in combination with other Ru-catalyst precursors was evaluated: however, only bis(2-methylallyl)(1,5-cyclooctadiene)ruthenium(ii) showed a similar reactivity, although with a slightly lower selectivity (Table S3†). The steric and electronic properties of Triphos seem to be peculiar as none of the other phosphines tested in combination with [Ru(acac)_3_] and Yb(OTf)_3_·H_2_O afforded a system active for amide reduction (Table S4†).

Several solvents other than tetrahydrofurane were screened in order to further improve the efficiency of the [Ru(acac)_3_]/Triphos/Yb(OTf)_3_·H_2_O system (Table S5†). In general, ether-based solvents and alcohols, with some exceptions, provided high conversions, but ethers afforded better selectivities. With isopropanol, ethylene glycol or 1,3-propandiol the main by-product was the one arising from solvent amination with aniline **4**. Both 2-methyl-terahydrofurane and methyl cyclopentyl ether, which have been listed as greener substitutes for THF,^14^ provided inferior selectivities in the desired *N*-benzylaniline **2** as to THF, which remained the solvent of choice for further tests.

The substrate scope for the reduction of several amides using the [Ru(acac)_3_]/Triphos/Yb(OTf)_3_·H_2_O system is reported in Table 3: the reactions were run under the optimised conditions, in THF at 150 °C and 5 bar H_2_ for a standard reaction time of 15 hours. For poorly reacting substrates, either the relative amount of catalyst was slightly increased or the reaction time was extended, thus improving both conversions and selectivities in the desired amines. Higher boiling solvents such as dioxane or ethylene glycol diethylether had to be used in such cases, instead of THF, which tended to partly condense outside the glass vials inside the autoclave over prolonged reaction times. In general, high conversions were obtained, with a few exceptions, while yields in the desired amines were more variable, depending on the structure of the amide. In any case, the only by-products were the alcohol and amine arising from hydrogenolysis of the amide.

**Table 3:** Substrate scope in the hydrogenation of amides catalyzed by [Ru(acac)_3_/Triphos/Yb(OTf)_3_·H_2_O] system

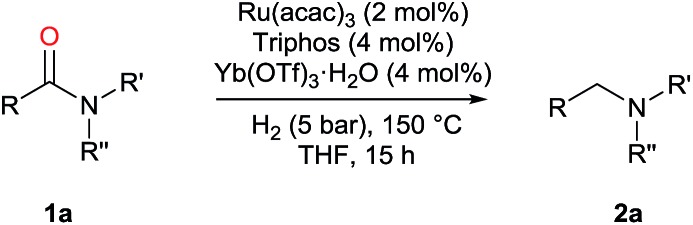					
Entry^*a*^	Amide **1a**	Conv.^*b*^ (%)	Amine **2a**	**2a** ^*b*^ (%)	Sel.^*b*^ (%)
1	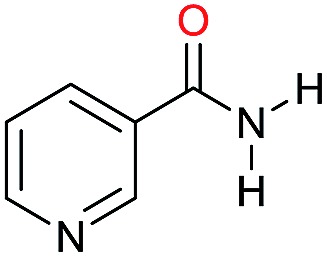	69	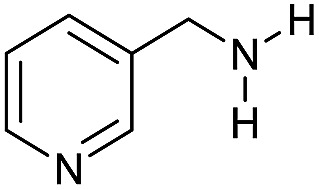	11	14
2	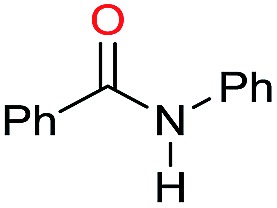	>99	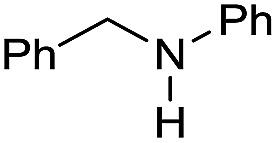	85 [80]	85
3	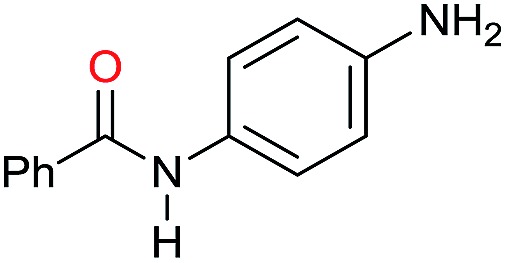	80	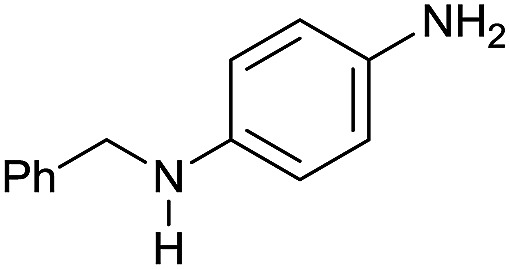	40	50
4	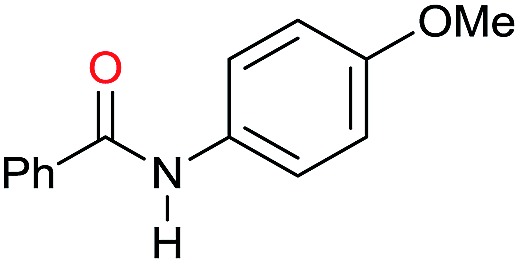	>99	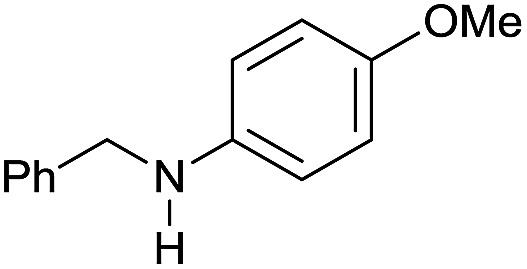	89 [79]	89
5	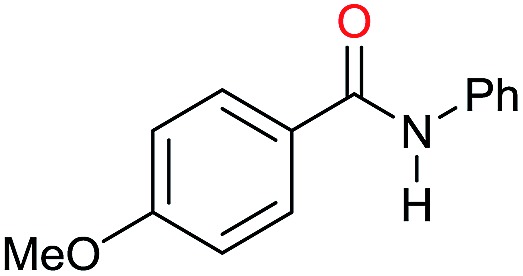	>99	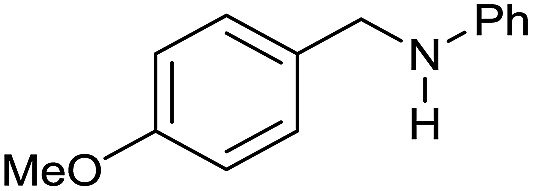	45	45
6^*c*^	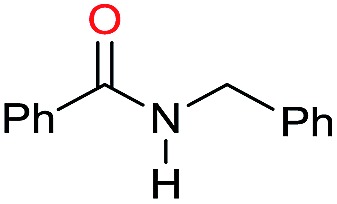	78	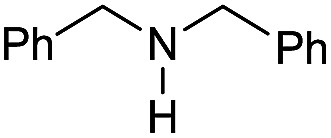	40	52
7^*d*^	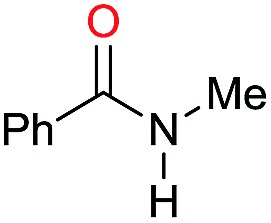	50	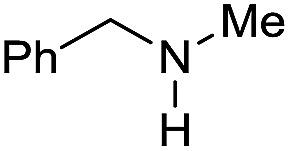	14	28
8^*e*^	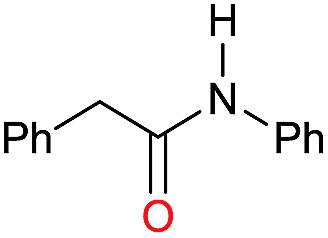	>99	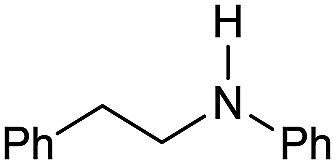	65	65
9	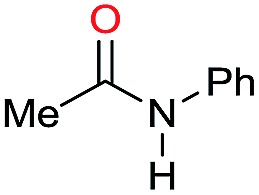	>99	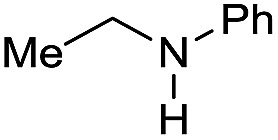	80	80
10	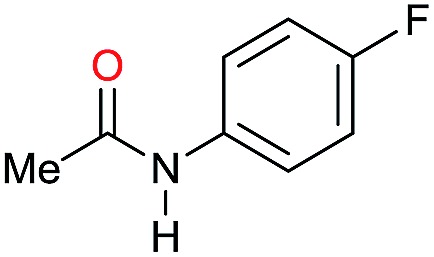	>99	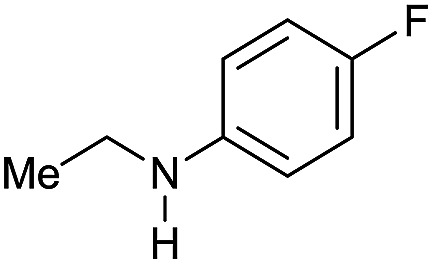	96 [82]	96
11^*f*^	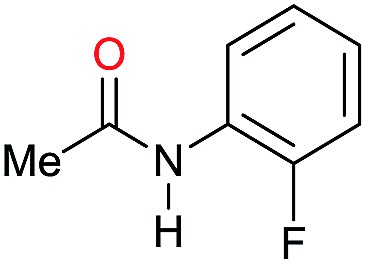	>99	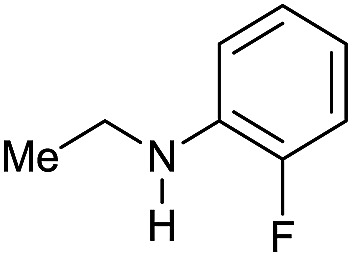	59	59
12^*g*^	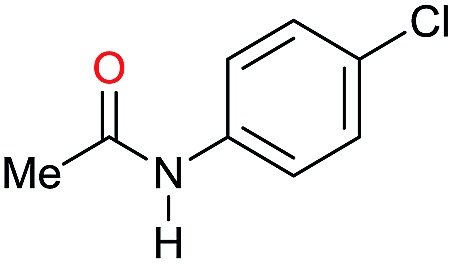	>99	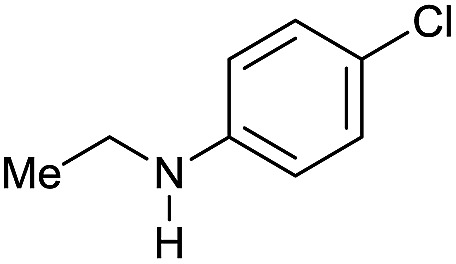	80 [70]	80
13^*f*^	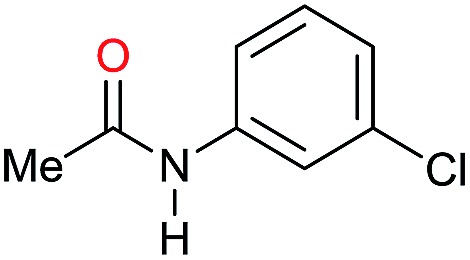	>99	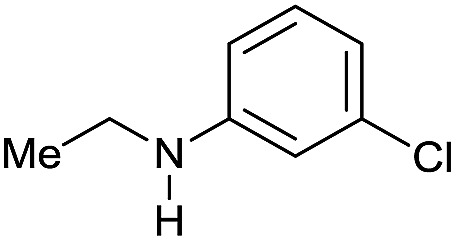	49	49
14^*g*^	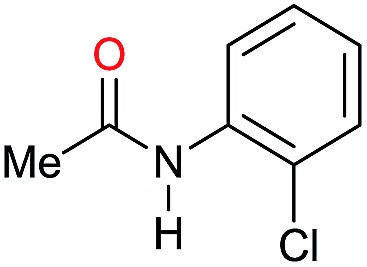	>99	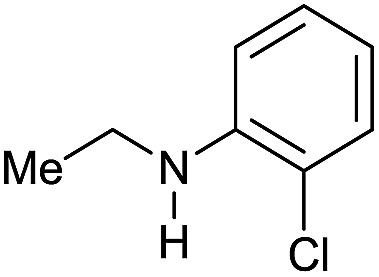	30	30
15	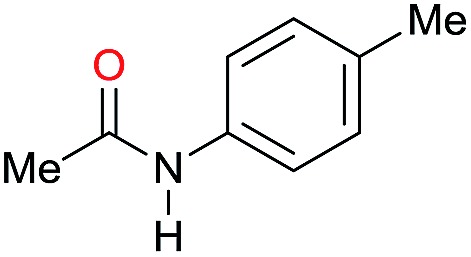	>99	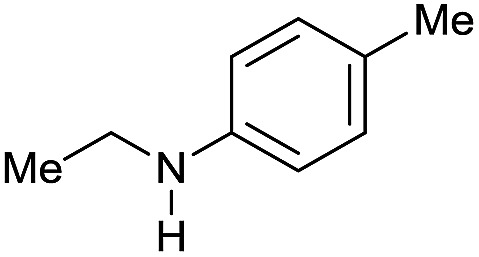	90	90
16	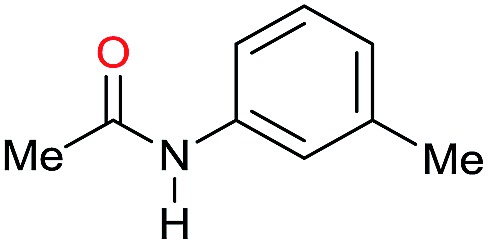	>99	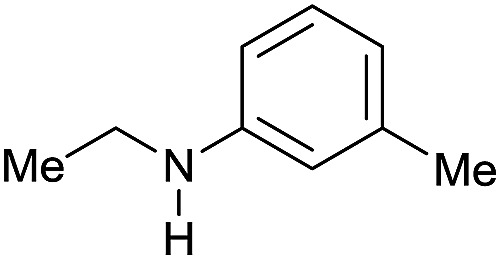	89 [82]	88
17^*h*^	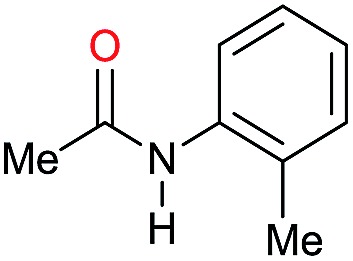	>99	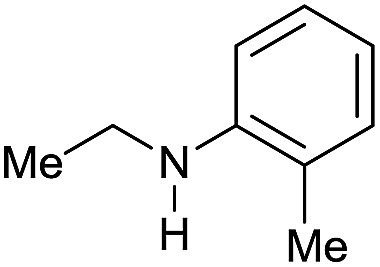	80	80
18	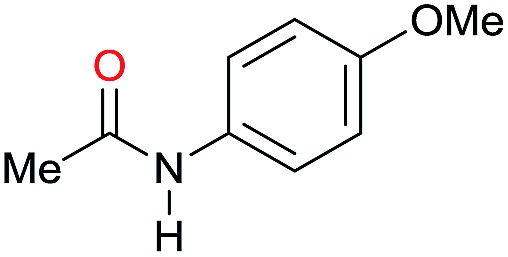	>99	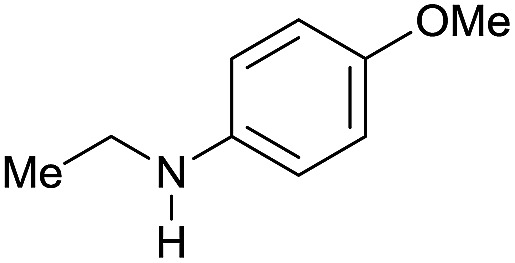	95 [88]	95
19^*i*^	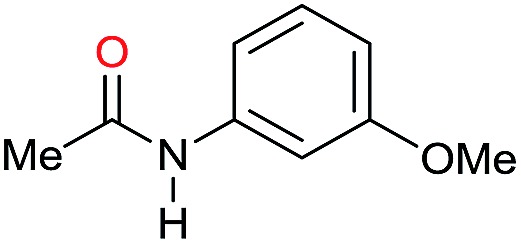	>99	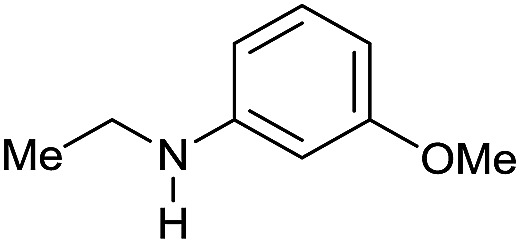	76	76
20	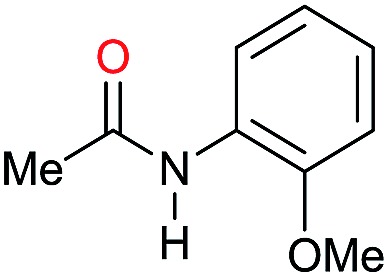	94	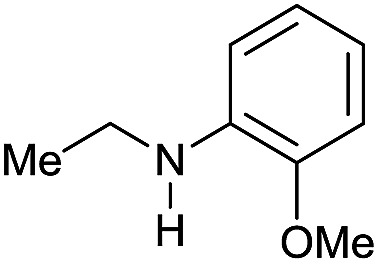	68	72
21	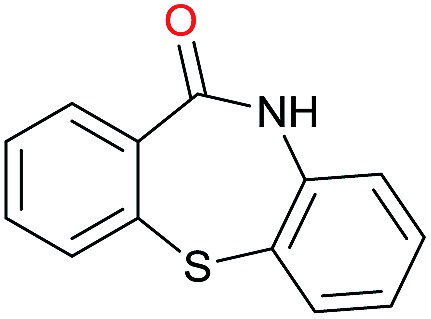	>99	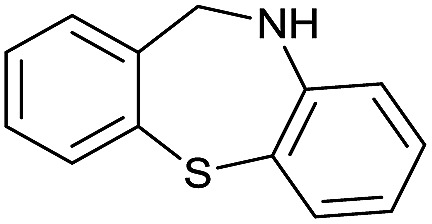	[86]	100
22^*d*^	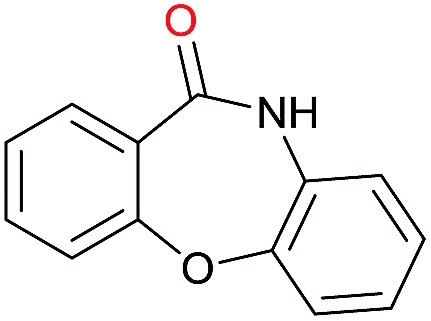	95	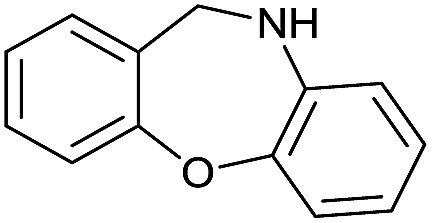	[84]	100
23^*i*^	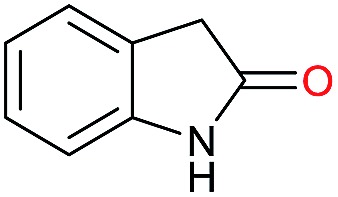	>99	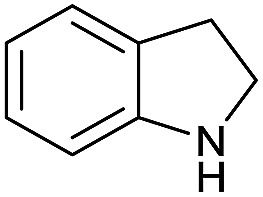	61	61
24^*c*^	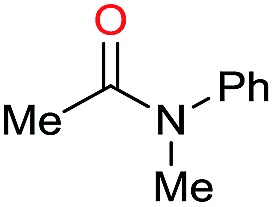	>99	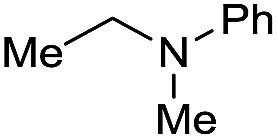	76	76
25^*c*^	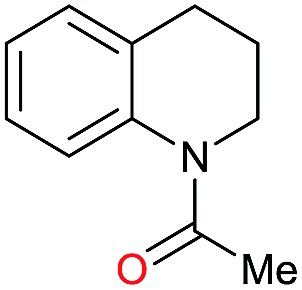	>99	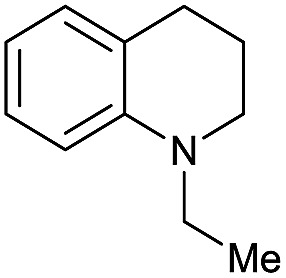	91 [85]	91
26^*j*^	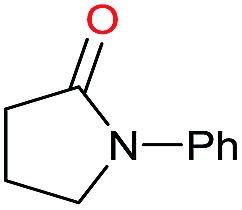	>99	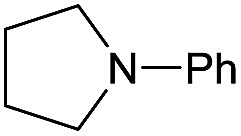	80 [70]	100
27^*c*^	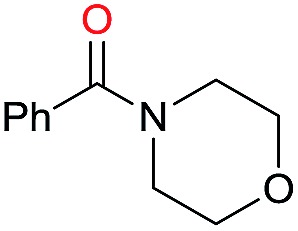	>99	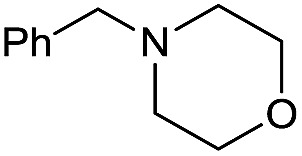	53	53
28^*d*^	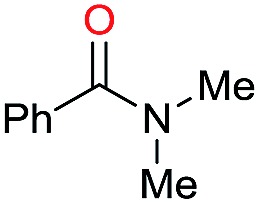	89	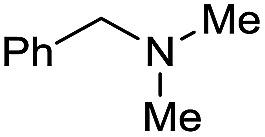	45	51
29^*c*^	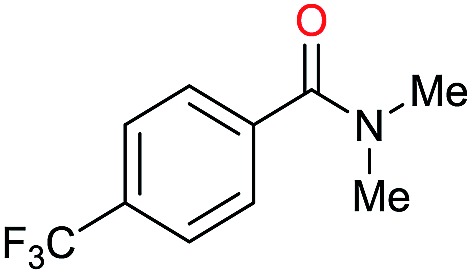	97	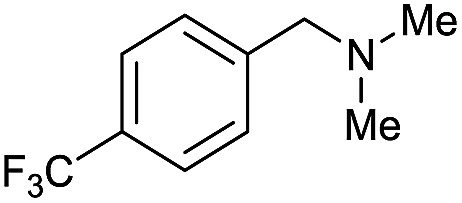	30	32
30^*d*^	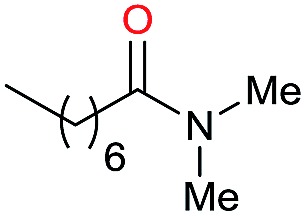	31	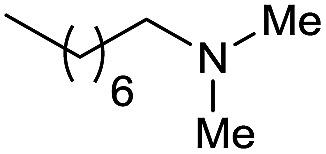	11	36

^*a*^Standard reaction conditions: amide (0.5 mmol), Ru(acac)_3_ (2 mol%), Triphos (4 mol%), Yb(OTf)_3_·H_2_O (4 mol%), THF (2 mL) and H_2_ (5 bar) at 150 °C, 15 h.

^*b*^Conversion of amide and yield of amine were calculated by GC using hexadecane as internal standard. The isolated yields, after column chromatography on silica gel, are reported between brackets. In all cases, only the alcohol and amine arising from the C–N bond cleavage in the parent amide were detected as by-products.

^*c*^Reaction conditions: Ru(acac)_3_ (6 mol%), Triphos (12 mol%), Yb(OTf)_3_·H_2_O (12 mol%), 1,4-dioxane (2 mL), 60 h.

^*d*^Reaction conditions: Ru(acac)_3_ (6 mol%), Triphos (12 mol%), Yb(OTf)_3_·H_2_O (12 mol%), THF (2 mL), 15 h.

^*e*^Reaction conditions: Ru(acac)_3_ (4 mol%), Triphos (8 mol%), Yb(OTf)_3_·H_2_O (8 mol%), 1,4-dioxane (2 mL), 60 h.

^*f*^Reaction conditions: Ru(acac)_3_ (6 mol%), Triphos (12 mol%), Yb(OTf)_3_·H_2_O (12 mol%), 1,4-dioxane (2 mL), 45 h.

^*g*^Reaction conditions: Ru(acac)_3_ (4 mol%), Triphos (8 mol%), Yb(OTf)_3_·H_2_O (8 mol%), THF (2 mL), 15 h.

^*h*^Reaction conditions: Ru(acac)_3_ (4 mol%), Triphos (8 mol%), Yb(OTf)_3_·H_2_O (8 mol%), 1,4-dioxane (2 mL), 45 h.

^*i*^Run at 50 bar of H_2_.

^*j*^Reaction conditions: Ru(acac)_3_ (6 mol%), Triphos (12 mol%), Yb(OTf)_3_·H_2_O (12 mol%), ethylene glycol diethylether (2 mL), 45 h.

Initially, the hydrogenation of the bio-relevant primary amide, nicotinamide, was achieved in moderate yields (Table 3, entry 1). Next, selected benzamides were tested (Table 3, entries 2–7) with *p*-benzanisidide (Table 3, entry 4) providing the highest yield, 89%. In agreement with previous reports,8*c* a phenyl substituent at nitrogen is beneficial and both activity and selectivity are progressively eroded going from *N*-phenyl to *N*-benzyl to *N*–Me (Table 3, entries 2, 6 and 7 respectively). Very good yields are obtained instead with the anilides of acetic (Table 3, entry 9, 80%) and phenylacetic acid (Table 3, entry 8, 65%). Several aryl-substituted acetanilides were then hydrogenated (Table 3, entries 10–20): all gave quantitative conversions and very good to excellent yields with the sole exceptions of 3-chloro- and 2-chloro acetanilide, whose amine yields were 49% and 30%, respectively. Overall, *para*-substituted acetanilides afforded a higher yield than the corresponding *meta*- and *ortho*-substituted ones and no direct correlation between the electronic nature of the substituents and amine yield became evident, although selectivity seems quite sensitive to sterics. Secondary amides containing functional groups such as ester (methyl-4-(phenylcarbamoyl)benzoate), nitro (4′-nitrobenzanilide) and alkenyl (*N*-phenyl acrylamide) were tested under optimized conditions affording low yields of the desired amine due to selectivity problems. In the case of substrates containing two amide groups the selectivity to the desired diamine was poor obtaining mixtures of over-alkylated amines. The present protocol was highly effective for the reduction of dibenzothiazepinone and dihydrodibenzoxazepinone, affording the corresponding heterocyclic amines in excellent isolated yields (Table 3, entries 21 and 22, 86 and 84%, respectively). Isoindoline was produced in 61% yield by hydrogenation of oxindole (Table 3, entry 23, 61%): while conversion was quantitative under standard reaction conditions, a higher pressure of hydrogen (50 bar) was required to maximise the selectivity in isoindoline at the expenses of 3*H*-indole, which is otherwise the main by-product. Several tertiary amides were reduced as well (Table 3, entries 24–30) giving access to *N*-substituted heterocycles in good (*N*-benzylmorpholine, 53%) to very good yields (*N*-ethyl-tetrahydroquinoline 91% and *N*-phenylpyrrolidine 80%). Although the yield of dimethyloctylamine was modest (Table 3, entry 30, 11%), the reduction with a homogeneous catalyst of the corresponding fully aliphatic tertiary amide, a notoriously challenging substrate, is the first ever reported.

**Scheme 2:**
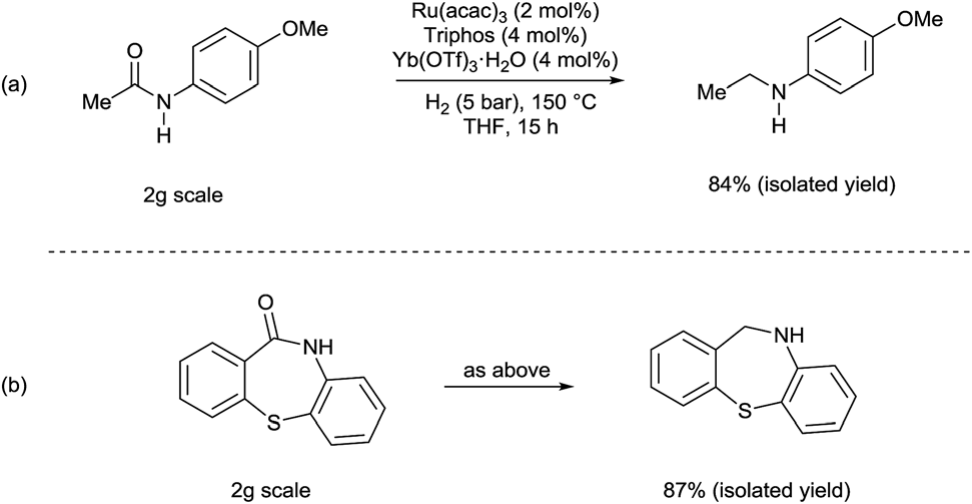
[Ru(acac)_3_/Triphos/Yb(OTf)_3_·H_2_O] catalyzed hydrogenation of amides: scale-up tests.

Although selected product amines were isolated with minor loss as to the GC yield, the feasibility of the synthetic protocol was further demonstrated by g-scale reactions of particular substrates. Indeed, *N*-ethyl-4-methoxyaniline (Scheme 2, eqn (a)) and 10,11-dihydro-5*H*-dibenzo[*b*,*e*]-[1,4]diazepine (Scheme 2, eqn (b)) were isolated in 84 and 87% yield, respectively.

Interestingly, during the assessment of the [Ru(acac)_3_]/Triphos/Yb(OTf)_3_·H_2_O system, it became clear that the selectivity in *N*-benzylaniline **2** varied over time increasing at higher conversions and that benzyl alcohol **3** and aniline **4** were essentially the only by-products in the reduction of benzanilide **1**.

**Fig. 2:**
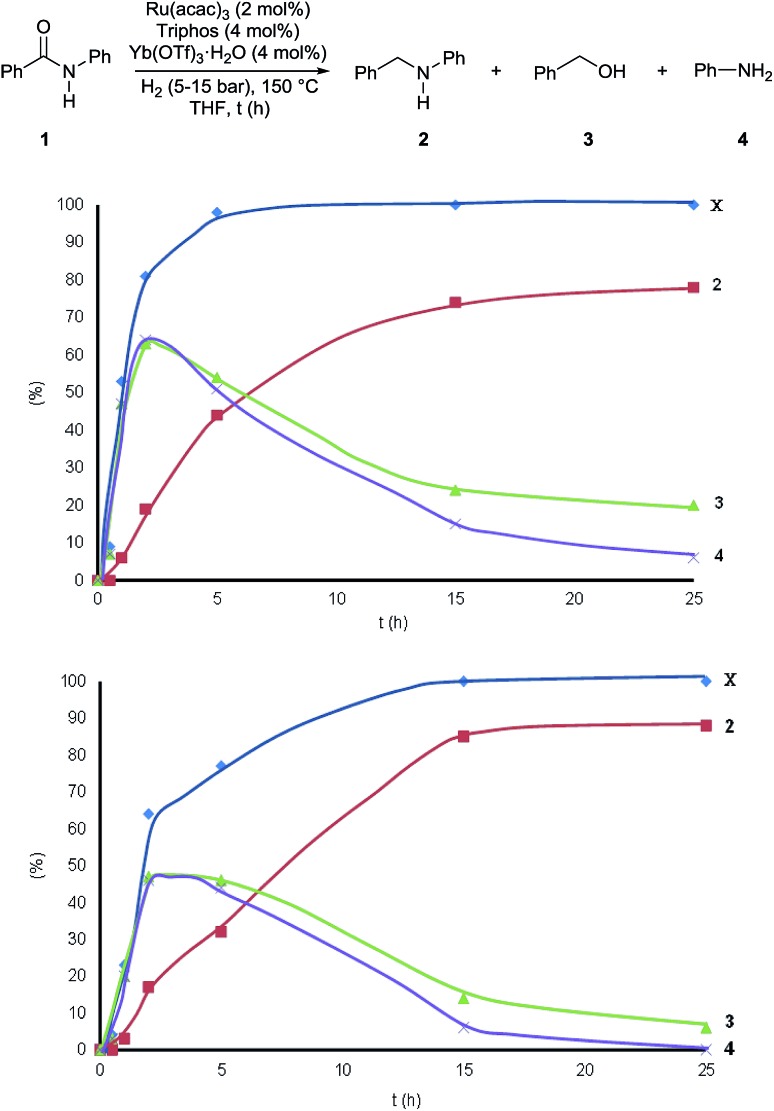
Variation of the substrate conversion and product yields during the hydrogenation of benzanilide **1** with the Ru/Triphos/Yb(OTf)_3_·H_2_O catalytic system. Reaction conditions: benzanilide 1 (100.6 mg, 0.5 mmol), Ru(acac)_3_ (2 mol%), Triphos (4 mol%), Yb(OTf)_3_·H_2_O (4 mol%), THF (2 mL) at 150 °C, H_2_ (15 bar, top graph), (5 bar, bottom graph). Conversion (X) of **1** and yields of **2**, **3**, and **4** were calculated by GC using hexadecane as internal standard. Variable amounts of *N*-phenylpyrrolidine (5–10%) were produced following Yb(OTf)_3_·H_2_O promoted ring-opening of THF.

Fig. 2 shows how the relative amounts of the substrate and the various products vary over time in the course of the reactions (Table S6†). To our surprise benzyl alcohol **3** and aniline **4** are the first observable products – intermediates – of benzanilide reduction. In the first 2 hours their relative amounts increase parallel to benzanilide conversion, which is quantitative after 5 hours, while product formation continues after that time! Interestingly, after reaching a maximum after about 3 h, the concentrations of benzyl alcohol **3** and aniline **4** drop as they are consumed to afford *N*-benzylaniline **2**.

It appears that benzyl alcohol **3** and aniline **4** are intermediates *en route* to the formation of *N*-benzylaniline **2**. They arise from collapse of the hemiaminal formed after the initial reduction of the amide carbonyl group. This is clearly in contrast with the established mechanistic proposal for heterogeneous or homogeneous amide reductions. In order to prove our assumption, a series of control experiments was carried out to shed light on the reaction pathway and the role of the Lewis acid. To begin with, a competitive experiment under the standard conditions was carried out by reacting benzanilide **1** with 3,5-dimethylbenzyl alcohol (Scheme S1†). As expected benzanilide was fully converted, but 70% of the resulting *N*-substituted aniline was the product of alkylation by 3,5-dimethylbenzyl alcohol. The preferential reaction with this alcohol indicates that reduction of benzanilide is slow as compared to the subsequent *N*-alkylation.

**Scheme 3:**
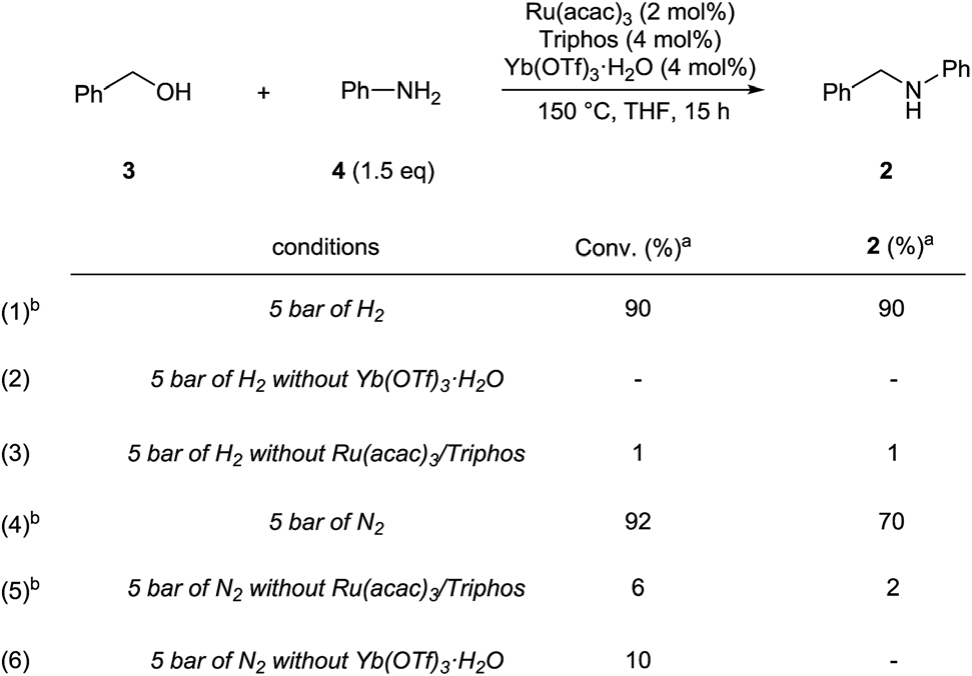
Control experiments showing the synergic combination of Ru(acac)_3_, Triphos and Yb(OTf)_3_·H_2_O in promoting the alkylation of aniline with benzyl alcohol (*via* hydrogen borrowing in the absence of hydrogen). ^a^ Conversion of **3** and yields of product **2** were calculated by GC using hexadecane as internal standard. ^b^ Variable amounts of *N*-phenylpyrrolidine (eqn (1) 7%, eqn (4) 4%, eqn (5) 6%) were produced following Yb(OTf)_3_·H_2_O promoted ring-opening of THF.

In agreement with our proposal, *N*-benzylaniline **2** is prepared in 90% yield from either the reaction of benzyl alcohol **3** (Scheme 3, eqn (1)) or benzaldehyde and aniline **4** under the previously optimized hydrogenation conditions (Scheme S2†).

As shown in Scheme 3 the synergistic combination of the [Ru(acac)_3_]/Triphos catalyst and Yb(OTf)_3_·H_2_O is needed for the amine alkylation as no conversion is observed otherwise (Scheme 3, eqn (2) and (3)). It is noteworthy that even without hydrogen the reaction between benzyl alcohol **3** and aniline **4** proceeded well to give *N*-benzylaniline **2** in 70% (Scheme 3, eqn (4)).

Examples have been reported in which metal triflates catalyze the direct amination of simple allylic, propargylic and benzylic alcohols through carbocation intermediates.^15^ Therefore, the possibility that, in the absence of hydrogen, *N*-benzylaniline **2** could be formed through direct *N*-alkylation of aniline **4** with benzyl alcohol **3** was taken into account. However Yb(OTf)_3_·H_2_O alone is not able to catalyse this reaction (Scheme 3, eqn (5)), neither is the [Ru(acac)_3_]/Triphos catalyst (Scheme 3, eqn (6)), hence both individual catalysts are required. Furthermore, the alkylation of aniline with 1-phenylethanol, a secondary benzylic alcohol which easily generates the stabilized carbocation, in the presence of hydrogen, provided only a poor 12% yield of the alkylated amine (Scheme S3†). Although in the absence of hydrogen conversion of the alcohol was higher, the main product though (45% yield) was in this case acetophenone. Significantly, control experiments were run using a primary aliphatic alcohol, 1-octanol, under identical reaction conditions (Schemes S4 and S5†). In fact, *N*-octylaniline was obtained in 92% yield in the presence of hydrogen, 55% without! Again in this case the combination of the [Ru(acac)_3_]/Triphos catalyst and Yb(OT)_3_·H_2_O is necessary. These results rule out a simple carbocation mechanism. Alkylation of aniline with benzyl alcohol is selective in that the product of double alkylation was never observed, not even when *N*-benzylaniline was reacted with extra benzyl alcohol (Scheme S7†).

The reactivity pattern of the alcohols, primary or secondary, benzylic or aliphatic, disproves a direct alkylation pathway. Instead, the control experiments in the absence of hydrogen indicate that the [Ru(acac)_3_]/Triphos/Yb(OTf)_3_·H_2_O system shows activity in the alkylation of aniline with an alcohol^16^ which occurs through a borrowing hydrogen pathway.^17^ The latter comprises the initial (i) oxidation (dehydrogenation) of the alcohol to form the corresponding aldehyde through hydrogen transfer to the metal catalyst,^18^ (ii) condensation of the aldehyde with the amine substrate to give an imine intermediate, and (iii) reduction of the imine by transfer of the hydrogen termini temporarily stored in the catalyst. The poor yields obtained with secondary alcohols reflects the inferior reactivity of ketones as to aldehydes in the reductive amination.^19^

**Fig. 3:**
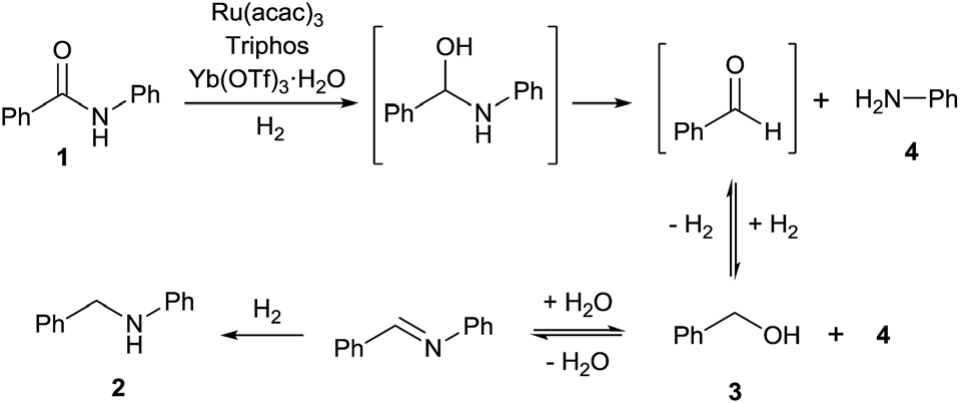
Proposed mechanism for the formation of the higher amine **2** from amide **1**.

Based on the experiments described above, a mechanism for the formation of amine **2** from amide **1** is proposed in Fig. 3. We believe that amide hydrogenation under the here described conditions initially leads to hydrogenolysis of the amide. Then, a borrowing hydrogen-type alkylation of the amine with the produced alcohol takes place. This new mechanistic insight opens the door for further catalyst improvements as well as new transformations, *e.g.* the reductive alkylation of amides as shown in Scheme S1.†

Although numerous ruthenium-based catalytic systems have been developed to date which promote the atom-economical and environmentally benign alkylation of amines with alcohols (water is the only by-product), the present system is quite different.^20^,^21^ In fact, the results of our control experiments, the recently reported Ru–Triphos catalyzed amination of alcohols with ammonia^22^ and the methylation of aromatic amines with formic acid as the sole carbon and hydrogen source^23^ add to the toolbox of useful synthetic transformation promoted by the Ru–Triphos system for the synthesis of amines, which includes their methylation with H_2_ and CO_2_ (ref. 24) and alkylation with carboxylic acids and hydrogen.^25^

It is clear that the possibility to achieve selective alkylation of amines with an alcohol by combining the Ru/Triphos system and Yb(OTf)_3_·H_2_O, as highlighted by the control experiments, deserves further investigations as these results represent the first examples of a reaction of this type which proceeds under acid catalysis and is currently being explored in our laboratories.

## Conclusions

The [Ru(acac)_3_]/Triphos catalytic system with Yb(OTf)_3_ constitutes an improved catalyst system for the hydrogenation of aliphatic and aromatic secondary and tertiary amides. Compared to previous work a significantly broader range of amides can be hydrogenated to the corresponding amines under milder conditions. No special care is needed and the reactions can be simply set in air. Control experiments indicate a different mechanism for this important transformation: after the initial reduction of the amide carbonyl group to the hemiaminal this intermediate collapses to give the alcohol and the non-alkylated amine. These compounds slowly produce the desired product *via* a hydrogen borrowing mechanism. The synergistic combination of Ru/Triphos and the metal triflate is necessary for both steps. Quantitative conversions were achieved with most of the tested substrates while selectivities still need to be improved for some of them. Further work is aimed to allow for hydrogenation under milder conditions in the presence of more demanding functionalized substrates, *e.g.* peptides. In the latter instance, because the only by-products are the alcohol and lower amine, investigations into the reductive amination step might serve to improve the system further on. Finally, it should be recognized that the [Ru(acac)_3_]/Triphos/Yb(OTf)_3_ system is competent for alcohol amination through hydrogen auto-transfer under acidic conditions.

## Experimental details

### General procedure for the hydrogenation of benzanilide (**1**)

A 8 mL glass vial containing a stirring bar was sequentially charged with benzanilide **1** (100.6 mg, 0.5 mmol), Ru(acac)_3_ (4.0 mg, 0.01 mmol), Triphos (12.5 mg, 0.02 mmol), Yb(OTf)_3_·H_2_O (12.8 mg, 0.02 mmol), *n*-hexadecane (50 mg) as an internal standard and THF (2 mL) as solvent. Afterwards, the reaction vial was capped with a septum equipped with a syringe needle and set in the alloy plate, which was then placed into a 300 mL autoclave. Once sealed, the autoclave was purged three times with 30 bar of hydrogen, then pressurized to 5–50 bar and placed into an aluminium block, which was preheated at 130–170 °C. After the desired reaction time (0.5–25 h), the autoclave was cooled in an ice bath, and the remaining gas was carefully released. Finally, the reaction mixture was diluted with ethyl acetate and analysed by GC.

## Supplementary Material

Supplementary information
