# Supplementary material for: Towards a general ruthenium-catalyzed hydrogenation of secondary and tertiary amides to amines
Source: Chem Sci. 2016 Feb 9;7(5):3432–42. doi: 10.1039/c5sc04671h (PMC6006866; doi:10.1039/c5sc04671h)

Electronic Supplementary Material (ESI) for Chemical Science.
This journal is © The Royal Society of Chemistry 2016

Electronic Supplementary Information

Towards a General Ruthenium-Catalyzed Hydrogenation of Secondary and Tertiary Amides to Amines

Jose R. Cabrero-Antonino,[a] Elisabetta Alberico,[a,b] Kathrin Junge,[a] Henrik Junge[a] and Matthias Beller[a*]

[a] Leibniz-Institut für Katalyse e.V. an der Universität Rostock, Albert-Einstein-Straße 29a, 18059 Rostock, Germany; Fax: (+49) 381-1281-5000

[b] Istituto di Chimica Biomolecolare, Consiglio Nazionale delle Ricerche, Tr. La Crucca 3, 07100 Sassari, Italy.

*E-mail: matthias.beller@catalysis.de

1. GENERAL INFORMATION

2. EXPERIMENTAL PROCEDURES

3. ADDITIONAL TABLES

Table S1. Hydrogenation of benzanilide (**1**) with the [Ru/Triphos/Hf(OTf)$_4$] system: Influence of the hydrogen pressure and temperature

Table S2. Hydrogenation of benzanilide (**1**) with the [Ru/Triphos/additive] system: screening of various Lewis and Brönsted acids as co-catalyst

Table S3. Hydrogenation of benzanilide (**1**) with the [Ru/Triphos/Yb(OTf)$_3$.H$_2$O] system: screening of different ruthenium precursors

Table S4. Hydrogenation of benzanilide (**1**) with the [Ru/L/Yb(OTf)$_3$.H$_2$O] system: screening of different phosphorus ligands

Table S5. Hydrogenation of benzanilide (**1**) with the [Ru/Triphos/Yb(OTf)$_3$.H$_2$O] system: Influence of the solvent

Table S6. Hydrogenation of benzanilide (**1**) with the [Ru/Triphos/Yb(OTf)$_3$.H$_2$O] system: yield/time profile at 5 and 15 bars H$_2$

4. ADDITIONAL SCHEMES

5. CHARACTERIZATION DATA OF THE ISOLATED PRODUCTS

6. REFERENCES

7. NMR SPECTRA OF THE ISOLATED PRODUCTS

1. GENERAL INFORMATION

All the chemicals were purchased and used without further purification. All hydrogenation reactions were set up in a 300 mL autoclave (PARR Instrument Company). In order to avoid unspecific reductions, all catalytic reactions were carried out in 8 mL glass vials, which were set in an alloy plate and placed inside the autoclave. The autoclave was then purged with 30 bar of hydrogen for three times before setting the pressure to the desired value. Conversions and yields of hydrogenation reactions were determined by GC-FID, HP 6890 with FID detector, column HP530 m x 250 mm x 0.25 μm. Mass spectra were recorded on a GC-MS Agilent 5973 Network equipped with a mass selective detector. NMR spectra were recorded using Bruker AV-300 (300 MHZ for 1H) and Bruker AV-400 (400 MHz for 1H) spectrometers. NMR chemical shifts are reported in parts per million (ppm) downfield from tetramethylsilane and were referenced to the residual proton resonance and the natural abundance ^{13}C resonance of the solvents. Coupling constants (*J*) are expressed in Hz. Abbreviations used in the reported NMR experiments: b, broad; s, singlet; d, doublet; t, triplet; q, quartet; m, multiplet. All measurements were carried out at room temperature unless otherwise stated. HRMS measurements of the all isolated products were performed using the electrospray ionization technique in an UPLC (ultra-pressure) equipment.

2. EXPERIMENTAL PROCEDURES

General procedure for the hydrogenation of amides: A 8 mL glass vial containing a stirring bar was sequentially charged with the amide (0.5 mmol), $Ru(acac)_3$ (2-6 mol%), triphos (4-12 mol%), $Yb(OTf)_3.H_2O$ (4-12 mol%), *n*-hexadecane (50 mg) as an internal standard and either THF, etilenglicol diethylether or 1,4-dioxane (2 mL) as solvent. Afterwards, the reaction vial was capped with a septum equipped with a syringe needle and set in the alloy plate, which was then placed into a 300 mL autoclave. Once sealed, the autoclave was purged three times with 30 bar of hydrogen, then pressurized to 5 bar and placed into an aluminium block, which was preheated at 150 °C. After the corresponding time (15-60 h), the autoclave was cooled in an ice bath, and the remaining gas was carefully released. Finally, the reaction mixture was diluted with ethyl acetate and analysed by GC. To determine the isolated yield of the amines, no internal standard was added and the reaction mixture was purified by silica gel column chromatography (*n*-heptane/ethyl acetate mixtures) to give the corresponding amines.

3. ADDITIONAL TABLES

Table S1. Hydrogenation of benzanilide (**1**) with [Ru/Triphos/$Hf(OTf)_4$] system: Influence of the hydrogen pressure and temperature

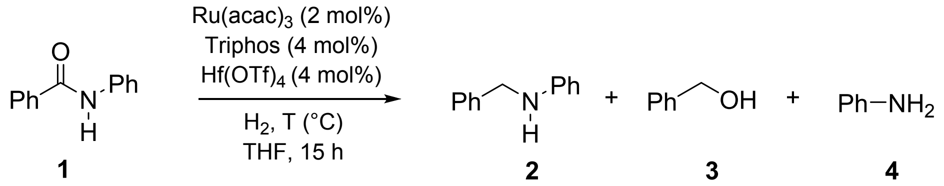

Entry[a]	T (°C)	H_2 (bar)	Conv. (%)[b]	**2** (%)[b]	**3** (%)[b]	**4** (%)[b]
1	170	50	100	46	45	18
2	150	50	100	42	51	37
3	130	50	96	30	67	64
4	150	25	100	57	39	26
**5**	**150**	**15**	**100**	**63**	**32**	**19**

[a] Standard reaction conditions: benzanilide **1** (100.6 mg, 0.5 mmol), Ru catalyst (2 mol%), triphos (4 mol%), $Hf(OTf)_4$ (4 mol%), THF (2 mL) and H_2 (15-50 bar) at 130-170 °C for 15 h. [b] Conversion of **1** and yields of **2**, **3**, and **4** were calculated by GC using hexadecane as internal standard. In some cases, variable amounts of *N*-phenylpyrrolidine (5-15%) were produced following acid promoted ring-opening of THF.

Table S2. Hydrogenation of benzanilide (**1**) with [Ru/Triphos/additive] system: screening of various Lewis and Bronsted acids as co-catalyst

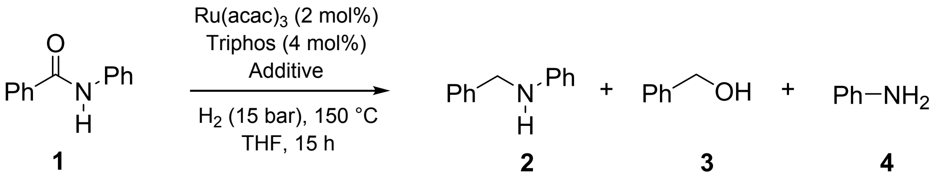

Entry[a]	Additive (mol%)	Conv. (%)[b]	**2** (%)[b]	**3** (%)[b]	**4** (%)[b]
1	$Mg(OTf)_2$ (4)	96	3	90	87
2	$Sc(OTf)_3$ (4)	100	71	27	20
3	$Mn(OTf)_2$ (4)	100	10	89	90
4	$Fe(OTf)_2$ (4)	97	51	43	42
5	$Ni(OTf)_2$ (4)	100	71	19	20
6	$Zn(OTf)_2$ (4)	100	65	32	28
7	$Al(OTf)_3$ (4)	100	72	27	22
8	$Ga(OTf)_3$ (4)	100	63	35	24
9	$In(OTf)_3$ (4)	100	61	38	29
10	$La(OTf)_3$ (4)	100	54	45	41
11	$Hf(OTf)_4$ (4)	100	63	32	19
12	$Ce(OTf)_3$ (4)	100	62	40	35
13	$Yb(OTf)_3.H_2O$ (2)	100	66	35	28
**14**	**$Yb(OTf)_3.H_2O$ (4)**	**100**	**74**	**24**	**15**
15	$Yb(OTf)_3.H_2O$ (6)	100	72	25	13
16	$MeSO_3H$ (1)	2	-	2	2
17	$MeSO_3H$ (2)	8	-	8	8
18	$MeSO_3H$ (4)	68	36	29	24
19	$HNTf_2$ (2)	98	3	77	66
20	$HNTf_2$ (4)	100	42	59	55
21	HOTf (16)	84	41	39	26

[a] Standard reaction conditions: benzanilide **1** (100.6 mg, 0.5 mmol), $Ru(acac)_3$ (2 mol%), triphos (4 mol%), additive (2-16 mol%), THF (2 mL) and H_2 (15 bar) at 150 °C during 15 h. [b] Conversion of **1** and yields of **2**, **3**, and **4** were calculated by GC using hexadecane as internal standard. In some cases, variable amounts of *N*-phenylpyrrolidine (5-15%) were produced following acid promoted ring-opening of THF.

Table S3. Hydrogenation of benzanilide (**1**) with [Ru/Triphos/$Yb(OTf)_3.H_2O$] system: screening of different ruthenium precursors

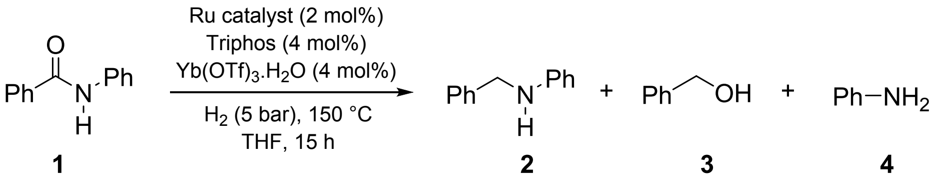

Entry[a]	Ru catalyst	Conv. (%)[b]	**2** (%)[b]	**3** (%)[b]	**4** (%)[b]
1	[$Ru(acac)_3$]	**100**	**85**	**14**	**6**
2	[Ru(COD)(methylallyl)$_2$]	100	78	21	18
3[c]	[{$RuCl_2$(benzene)}$_2$]	-	-	-	-
4	[$Ru(PPh_3)_3(CO)H_2$]	4	3	1	1
5	PhMe.[$Ru(PPh_3)_3ClH$]	-	-	-	-
6	[$Ru(dmso)_4Cl_2$]	-	-	-	-
7[c]	[Ru(Cp)(p-cymene)]PF_6	-	-	-	-

[a] Standard reaction conditions: benzanilide **1** (100.6 mg, 0.5 mmol), Ru catalyst (2 mol%), triphos (4 mol%), $Yb(OTf)_3.H_2O$ (4 mol%), THF (2 mL) and H_2 (5 bar) at 150 °C during 15 h. [b] Conversion of **1** and yields of **2**, **3**, and **4** were calculated by GC using hexadecane as internal standard. In some cases, variable amounts of *N*-phenylpyrrolidine (5-10%) were produced following $Yb(OTf)_3{\cdot}H_2O$ promoted ring-opening of THF. [c] The reaction was carried out with 1 mol% of Ru catalyst (2 mol% Ru).

Table S4. Hydrogenation of benzanilide (**1**) with [Ru/L/Yb(OTf)$_3$.H$_2$O] system: screening of different phosphorus ligands

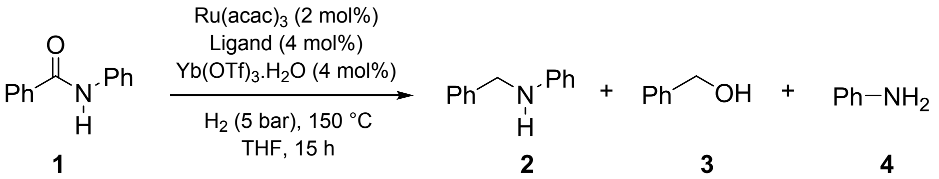

Entry[a]	Ligand	Conv. (%)[b]	**2** (%)[b]	**3** (%)[b]	**4** (%)[b]
1	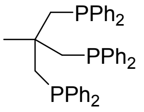	**100**	**85**	**14**	**6**
2	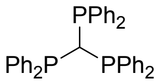	-	-	-	-
3	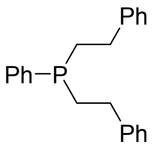	-	-	-	-
4[c]	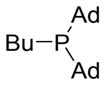	53	-	-	-
5	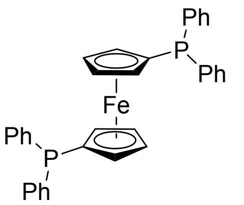	2	-	-	-
6	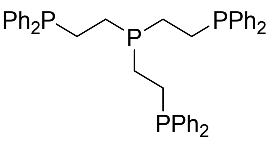	-	-	-	-
7	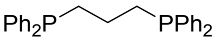	-	-	-	-
8	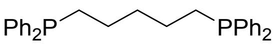	1	-	-	-
9	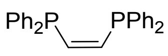	-	-	-	-
10	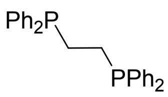	-	-	-	-

[a] Standard reaction conditions: benzanilide **1** (100.6 mg, 0.5 mmol), Ru(acac)$_3$ (2 mol%), ligand (4 mol%), Yb(OTf)$_3$.H$_2$O (4 mol%), THF (2 mL) and H$_2$ (5 bar) at 150 °C during 15 h. [b] Conversion of **1** and yields of **2**, **3**, and **4** were calculated by GC using hexadecane as internal standard. In some cases, variable amounts of *N*-phenylpyrrolidine (5-10%) were produced following Yb(OTf)$_3$·H$_2$O promoted ring-opening of THF. [c] The main products observed were hydrogenation ring products. (Ad = adamantyl).

Table S5. Hydrogenation of benzanilide (**1**) with [Ru/Triphos/Yb(OTf)$_3$.H$_2$O] system: Influence of the solvent

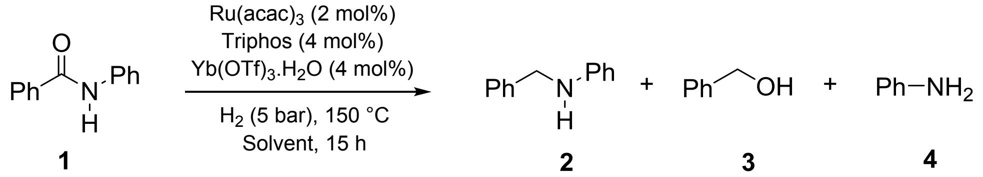

Entry[a]	Solvent	Conv. (%)[b]	**2** (%)[b]	**3** (%)[b]	**4** (%)[b]
**1**	**THF**	**100**	**85**	**14**	**6**
2	2-Me-THF	96	65	27	24
3	MCPE	62	32	32	31
4	Tetrahydropyrane	100	79	16	9
5	1,4-Dioxane	100	67	33	29
6	Isopropanol	100	55	36	-
7	Trifluoroethanol	79	28	48	52
8	t-Amyl alcohol	26	1	25	25
9	Ethylene glycol	100	19	82	-
10	Propanediol	100	6	94	7
11	Ethylene glycol diethylether	66	19	44	35
12[c]	Ethylene glycol diethylether	100	68	29	7
13	Toluene	53	36	21	19

[a] Standard reaction conditions: benzanilide (0.5 mmol), Ru(acac)$_3$ (2 mol%), triphos (4 mol%), Yb(OTf)$_3$.H$_2$O (4 mol%), solvent (2 mL) and H$_2$ (5 bar) at 150 °C during 15 h. [b] Conversion of **1** and yields of products **2**, **3** and **4** were calculated by GC using hexadecane as internal standard. [c] Run at 45 h.

Table S6. Hydrogenation of benzanilide (**1**) with [Ru/Triphos/Yb(OTf)$_3$.H$_2$O] system: yield/time profile at 5 and 15 bars H$_2$

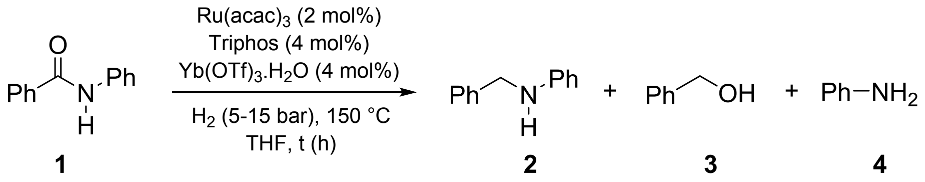

Entry[a]	H_2 (bar)	t (h)	Conv. (%)[b]	**2** (%)[b]	**3** (%)[b]	**4** (%)[b]	Sel. **2** (%)
1	15	0.5	9	-	7	7	-
2		1	53	6	47	47	11
3		2	81	19	63	64	23
4		5	98	44	54	51	45
5		15	100	74	24	15	74
6		25	100	78	20	6	78
7	5	0.5	4	-	3	3	-
8		1	23	3	20	20	5
9		2	64	17	47	46	27
10		5	77	32	46	44	42
11		15	100	85	14	6	85
12		25	100	88	6	-	88

[a] Standard reaction conditions: benzanilide **1** (100.6 mg, 0.5 mmol), Ru catalyst (2 mol%), triphos (4 mol%), Yb(OTf)$_3$.H$_2$O (4 mol%), THF (2 mL) and H_2 (5 or 15 bar) at 150 °C during 0.5-25 h. [b] Conversion of **1** and yields of **2**, **3**, and **4** were calculated by GC using hexadecane as internal standard. Variable amounts of *N*-phenylpyrrolidine (5-10%) were produced following Yb(OTf)$_3$·H$_2$O promoted ring-opening of THF.

4. ADDITIONAL SCHEMES

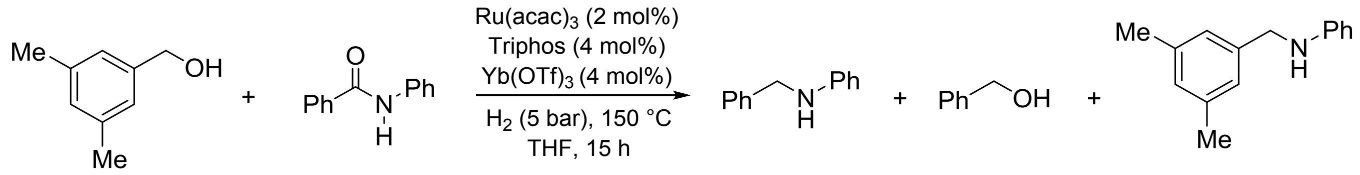

1 Full conversion (ref. to **1**) **2** (29%) **3** (69%) (70%)

Scheme S1. Reaction control experiment using 3,5-dimethylbenzyl alcohol and benzanilide **1** as starting materials. Conversion of **1** and yields of the products were calculated by GC using hexadecane as internal standard.

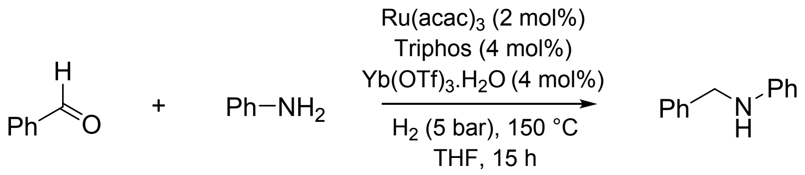

4 (1.5 eq) Full conversion **2** (86%)

Scheme S2. Reaction control experiment using benzaldehyde and aniline as starting materials. Conversion of benzaldehyde and yield of product **2** were calculated by GC using hexadecane as internal standard.

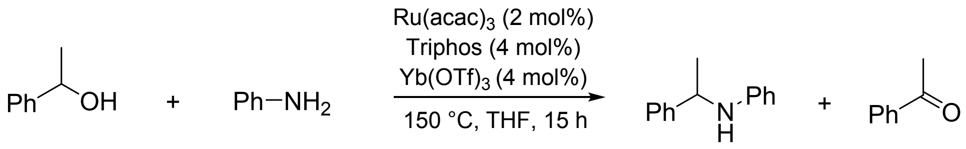

1 eq 1.5 eq

conditions	Conv. (%)[a]	(%)[a]	(%)[a]
5 bar of H_2	20	12	-
5 bar of N_2	61	12	45

Scheme S3. Reaction control experiments using 1-phenylethanol and aniline as starting materials. [a] Conversion of 1-phenylethanol and yield of the products were calculated by GC using hexadecane as internal standard.

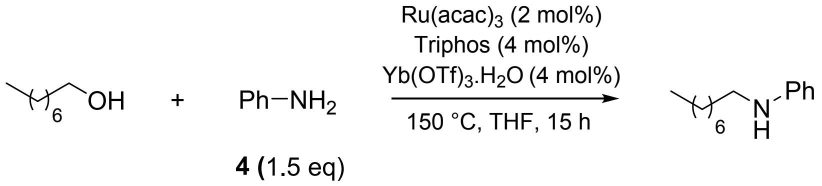

conditions	Conv. (%)[a]	(%)[a]
5 bar of H_2	95	92
5 bar of H_2 without $Yb(OTf)_3.H_2O$	-	-
5 bar of H_2 without $Ru(acac)_3$/Triphos	-	-
5 bar of N_2	90	55
5 bar of N_2 without $Yb(OTf)_3.H_2O$	-	-
5 bar of N_2 without $Ru(acac)_3$/Triphos	6	-

Scheme S4. Reaction control experiments using octanol and aniline as starting materials. [a] Conversion of octanol and yield of the product were calculated by GC using hexadecane as internal standard. In some cases, variable amounts of *N*-phenylpyrrolidine (5-10%) were produced following $Yb(OTf)_3{\cdot}H_2O$ promoted ring-opening of THF.

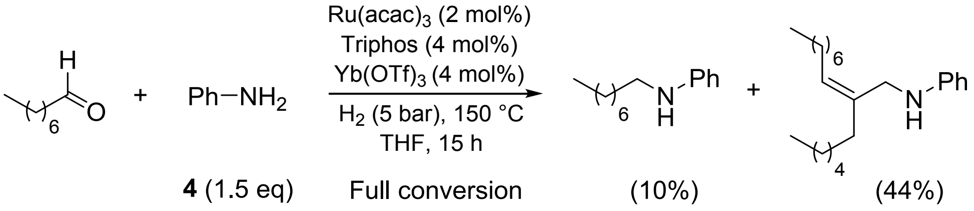

Scheme S5. Reaction control experiment using octanal and aniline as starting materials. Conversion of octanal and yield of the products were calculated by GC using hexadecane as internal standard.

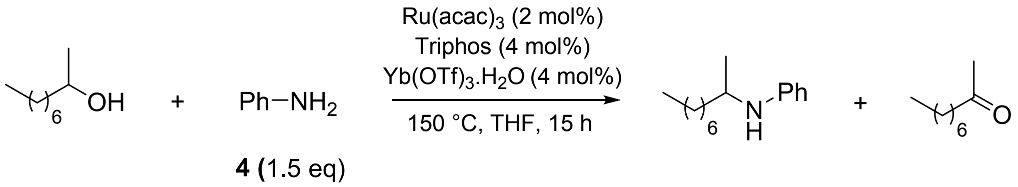

conditions	Conv. (%)[a]	(%)[a]	(%)[a]
5 bar of H_2	5	-	-
5 bar of H_2 without $Yb(OTf)_3.H_2O$	-	-	-
5 bar of H_2 without $Ru(acac)_3$/Triphos	-	-	-
5 bar of N_2	49	-	45
5 bar of N_2 without $Yb(OTf)_3.H_2O$	-	-	-
5 bar of N_2 without $Ru(acac)_3$/Triphos	-	-	-

Scheme S6. Reaction control experiments using 2-nonanol and aniline as starting materials. [a] Conversion of 2-nonanol and yield of the products were calculated by GC using hexadecane as internal standard. In some cases, variable amounts of *N*-phenylpyrrolidine (5-10%) were produced following $Yb(OTf)_3{\cdot}H_2O$ promoted ring-opening of THF.

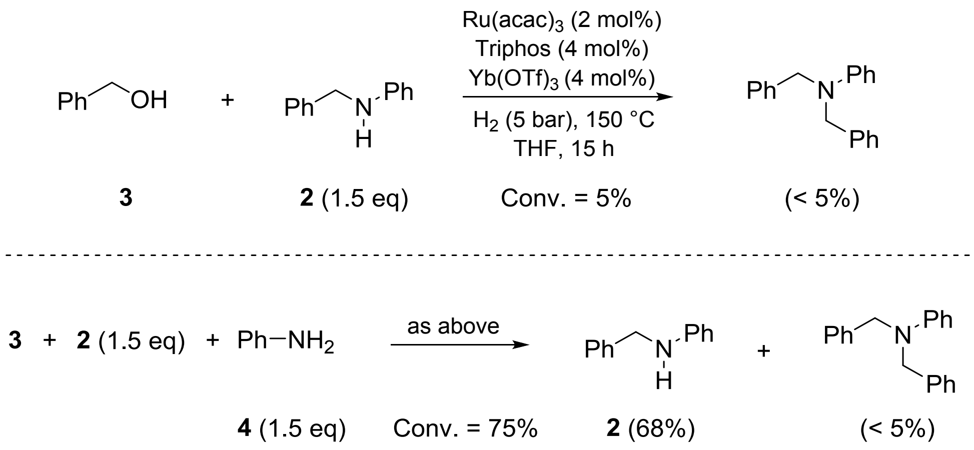

Scheme S7. Competitive experiments using N-benzylaniline **2** as starting material. Conversion of **3** and yield of the products were calculated by GC using hexadecane as internal standard.

5. CHARACTERIZATION DATA OF THE ISOLATED PRODUCTS

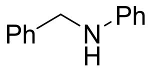

N-benzylaniline[1]. Isolated yield: 80%. GC-MS (m/z, $M^{+\cdot}$ 183), major peaks found: 183 (75%), 106 (20%), 91 (100%), 77 (19%), 65 (16%), 51 (9%). (The NMR spectrum is consistent with the reported data). ^{1}H NMR (300 MHz, $CDCl_3$): 7.32-7.15 (m, 5H), 7.14-7.03 (m, 2H), 6.63 (tt, J = 7.3, 1.1, 1H), 6.58-6.51 (m, 2H), 4.23 (s, 2H), 3.92 (bs, NH). ^{13}C NMR (75 MHz, $CDCl_3$): 148.25 (C), 139.54 (C), 129.38 (2xCH), 128.75 (2xCH), 127.63 (2xCH), 127.34 (CH), 117.68 (CH), 112.96 (2xCH), 48.44 (N-CH_2).

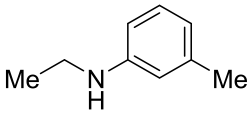

N-ethyl-3-methylaniline[2]. Isolated yield: 82%. GC-MS (m/z, $M^{+\cdot}$ 135), major peaks found: 135 (39%), 120 (100%), 91 (16%), 77 (8%), 65 (8%). (The NMR spectrum is consistent with the reported data). ^{1}H NMR (300 MHz, $CDCl_3$): 7.12-7.04 (m, 1H), 6.55 (d, J = 7.2, 1H), 6.45 (d, J = 6.4, 2H), 3.17 (q, J = 7.1, 2H), 2.30 (s, 3H), 1.26 (t, J = 7.1, 3H). ^{13}C NMR (75 MHz, $CDCl_3$): 148.56 (C), 139.09 (C), 129.21 (CH), 118.34 (CH), 113.70 (CH), 110.11 (CH), 38.66 (N-CH_2), 21.76 (CH_3), 15.05 (CH_3).

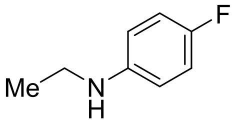

N-ethyl-4-fluoroaniline[3]. Isolated yield: 82%. GC-MS (m/z, $M^{+\cdot}$ 139), major peaks found: 139 (30%), 124 (100%), 95 (12%), 83 (9%). (The NMR spectrum is consistent with the reported data).^{1}H NMR (300 MHz, $CDCl_3$): 6.95-6.84 (m, 2H), 6.59-6.50 (m, 2H), 3.12 (q, J = 7.1, 2H), 1.25 (t, J = 7.1, 3H). ^{13}C NMR (75 MHz, $CDCl_3$): 155.95 (d, $J^{1}_{C\text{-}F}$ = 234.6, C), 144.81 (C), 115.75 (d, $J^{2}_{C\text{-}F}$ = 22.2), 113.78 (d, $J^{3}_{C\text{-}F}$ = 7.4), 39.39 (N-CH_2), 14.38 (CH_3). ^{19}F NMR (282 MHz, $CDCl_3$): -124.65-(-131.79) (m, 1F).

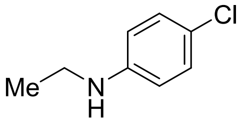

N-ethyl-4-chloroaniline[4]. Isolated yield: 70%. GC-MS (m/z, $M^{+\cdot}$ 155), major peaks found: 157 (12%), 155 (36%), 142 (33%), 140 (100%), 111 (8%), 77 (8%). (The NMR spectrum is consistent with the reported data). ^{1}H NMR (300 MHz, $CDCl_3$): 7.11 (d, J = 7.1, 2H), 6.52 (d, J = 7.1, 2H), 3.55 (bs, NH), 3.12 (q, J = 7.1, 2H), 1.25 (t, J = 7.1, 3H). ^{13}C NMR (75 MHz, $CDCl_3$): 147.09 (C), 129.13 (2xCH), 121.79 (C), 113.87 (2xCH), 38.69 (N-CH_2), 14.85 (CH_3).

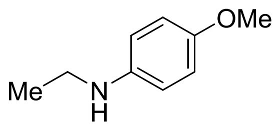

N-ethyl-4-fluoroaniline[1]. Isolated yield: 82%. GC-MS (m/z, $M^{+\cdot}$ 151), major peaks found: 151 (50%), 136 (100%), 121 (8%), 108 (15%), 80 (10%). (The NMR spectrum is consistent with the reported data). ^{1}H NMR (300 MHz, $CDCl_3$): 6.80 (d, J = 8.9, 2H), 6.60 (d, J = 8.9, 2H), 3.76 (s, 3H), 3.25 (bs, NH), 3.12 (q, J = 7.1, 2H), 1.25 (t, J = 7.1, 3H). ^{13}C NMR (75 MHz, $CDCl_3$): 152.15 (C), 142.82 (C), 114.97 (2xCH), 14.23 (2xCH), 55.91 (O-CH_3), 39.57 (N-CH_2), 15.09 (CH_3).

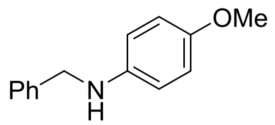

N-benzyl-4-methoxyaniline[1]. Isolated yield: 79%. GC-MS (m/z, $M^{+\cdot}$ 213), major peaks found: 213 (100%), 198 (10%), 136 (9%), 122 (78%), 91 (65%), 65 (12%). (The NMR spectrum is consistent with the reported data). ^{1}H NMR (300 MHz, $CDCl_3$): 7.33-7.10 (m, 5H), 6.75-6.63 (m, 2H), 6.57-6.45 (m, 2H), 4.18 (s, 2H), 3.64 (s, 3H), 3.40 (bs, NH). ^{13}C NMR (75 MHz, $CDCl_3$): 152.28 (C), 142.55 (C), 139.79 (C), 128.70 (2xCH), 127.65 (2xCH), 127.27 (CH), 115.00 (2xCH), 114.21 (2xCH), 55.89 (O-CH_3), 49.33 (N-CH_2). HRMS (ESI) [M^+; calculated for $C_{14}H_{15}ON$: 213.1148] found m/z 213.1149.

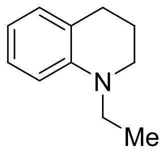

1-ethyl-1,2,3,4-tetrahydroquinoline[5]. Isolated yield: 80%. GC-MS (m/z, $M^{+\cdot}$ 161), major peaks found: 161 (42%), 146 (100%), 130 (20%), 118 (15%), 91 (14%), 77 (10%). (The NMR spectrum is consistent with the reported data). ^{1}H NMR (300 MHz, $CDCl_3$): 7.12-7.03 (m, 1H), 7.00-6.94 (m, 1H), 6.66-6.55 (m, 2H), 3.37 (q, J = 7.1, 2H), 3.28 (q, J = 5.7, 2H), 2.78 (t, J = 6.4, 2H), 2.06-1.91 (m, 2H), 1.17 (t, J = 7.1, 3H). ^{13}C NMR (75 MHz, $CDCl_3$): 145.11 (C), 129.27 (CH), 127.18 (CH), 122.55 (C), 115.46 (CH), 110.64 (CH), 48.50 (CH_2), 45.42 (CH_2), 28.30 (CH_2), 22.41 (CH_2), 10.91 (N-CH_3).

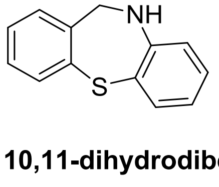

10,11-dihydrodibenzo[b,f][1,4]thiazepine[6]. Isolated yield: 86%. GC-MS (m/z, $M^{+\cdot}$ 212), major peaks found: 212 (100%), 197 (11%), 180 (30%), 152 (9%). (The NMR spectrum is consistent with the reported data). ^{1}H NMR (300 MHz, $CDCl_3$): 7.56-7.49 (m, 1H), 7.31-7.14 (m, 4H), 6.91 (ddd, J = 8.2, 7.3, 1.6, 1H), 6.56 (td, J = 7.5, 1.3, 1H), 6.36 (dd, J = 8.1, 1.3, 1H), 4.80 (s, 2H), 3.74 (bs, NH). ^{13}C NMR (75 MHz, $CDCl_3$): 146.93 (C), 142.95 (C), 137.17

(C), 132.42 (CH), 131.50 (CH), 128.69 (CH), 128.43 (CH), 128.32 (CH), 128.03 (CH), 118.37 (CH), 117.71 (CH), 116.19 (C), 48.98 (N-CH_2). HRMS (ESI) [M^+; calculated for $C_{13}H_{10}NS$: 212.0528] found m/z 212.0526.

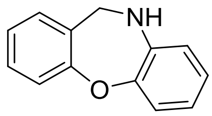

10,11-dihydrodibenzo[b,f][1,4]oxazepine[6]. Isolated yield: 84%. GC-MS (m/z, $M^{+\cdot}$ 197), major peaks found: 197 (100%), 168 (40%), 139 (8%), 120 (30%). (The NMR spectrum is consistent with the reported data). ^{1}H NMR (300 MHz, $CDCl_3$): 7.20-7.11 (m, 1H), 7.07 (m, 2H), 7.03-6.93 (m, 2H), 6.75 (td, J = 7.9, 1.6, 1H), 6.58 (td, J = 7.9, 1.6, 1H), 6.44 (dd, J = 7.9, 1.6, 1H), 4.36 (s, 1H), 3.59 (bs, NH). ^{13}C NMR (75 MHz, $CDCl_3$): 158.44 (C), 144.93 (C), 138.90 (C), 131.82 (C), 129.13 (CH), 128.12 (CH), 124.50 (CH), 124.36 (CH), 122.11 (CH), 120.63 (CH), 119.32 (CH), 118.67 (CH), 46.97 (N-CH_2). HRMS (ESI) [M^+H^+; calculated for $C_{13}H_{11}NO$: 198.0913] found m/z 198.0913.

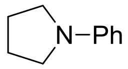

1-phenylpyrrolidine[1]. Isolated yield: 70%. GC-MS (m/z, $M^{+\cdot}$ 146), major peaks found: 146 (100%), 119 (8%), 104 (16%), 91 (45%), 77 (35%), 51 (10%). (The NMR spectrum is consistent with the reported data). ^{1}H NMR (300 MHz, $CDCl_3$): 7.40-7.29 (m, 2H), 6.79 (t, J = 7.3, 1H), 6.69 (d, J = 7.8, 2H), 3.42-3.35 (m, 4H), 2.19-1.96 (m, 4H). ^{13}C NMR (75 MHz, $CDCl_3$): 148.03 (C), 129.17 (2xCH), 115.43 (CH), 111.71 (2xCH), 47.63 (2xCH_2), 25.53 (2xCH_2).

6. REFERENCES

(1) Zou, Q.; Wang, C.; Smith, J.; Xue, D.; Xiao, J. *Chem. Eur. J.* **2015**, *21*, 9656.

(2) Nacario, R.; Kotakonda, S.; Fouchard, D. M. D.; Tillekeratne, L. M. V.; Hudson, R. A. *Org. Lett.* **2005**, *7*, 471.

(3) Ikawa, T.; Fujita, Y.; Mizusaki, T.; Betsuin, S.; Takamatsu, H.; Maegawa, T.; Monguchi, Y.; Sajiki, H. *Org. Biomol. Chem.* **2012**, *10*, 293.

(4) Garcia Ruano, J. L.; Parra, A.; Aleman, J.; Yuste, F.; Mastranzo, V. M. *Chem. Commun.* **2009**, 404.

(5) Abarca, B.; Adam, R.; Ballesteros, R. *Org. Biomol. Chem.*, **2012**, *10*, 1826.

(6) Sum, F.-W.; Dusza, J.; Delos Santos, E.; Grosu, G.; Reich, M.; Du, X.; Albright, J. D.; Chan, P.; Coupet, J.; Ru, X.; Mazandarani, H.; Saunders, T. *Bioorg. Med. Chem. Lett.* **2003**, *13*, 2195.

7. NMR SPECTRA OF THE ISOLATED PRODUCTS

^{1}H NMR

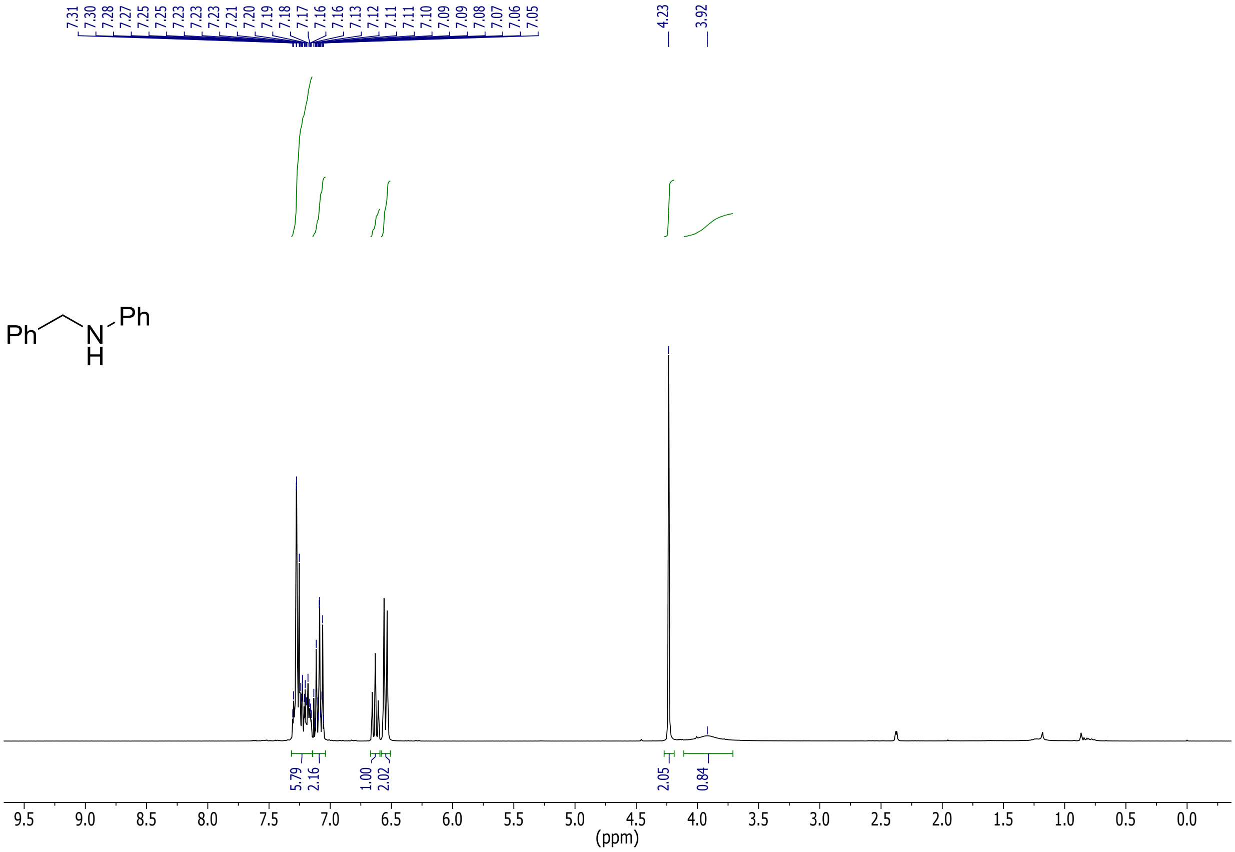

^{13}C NMR

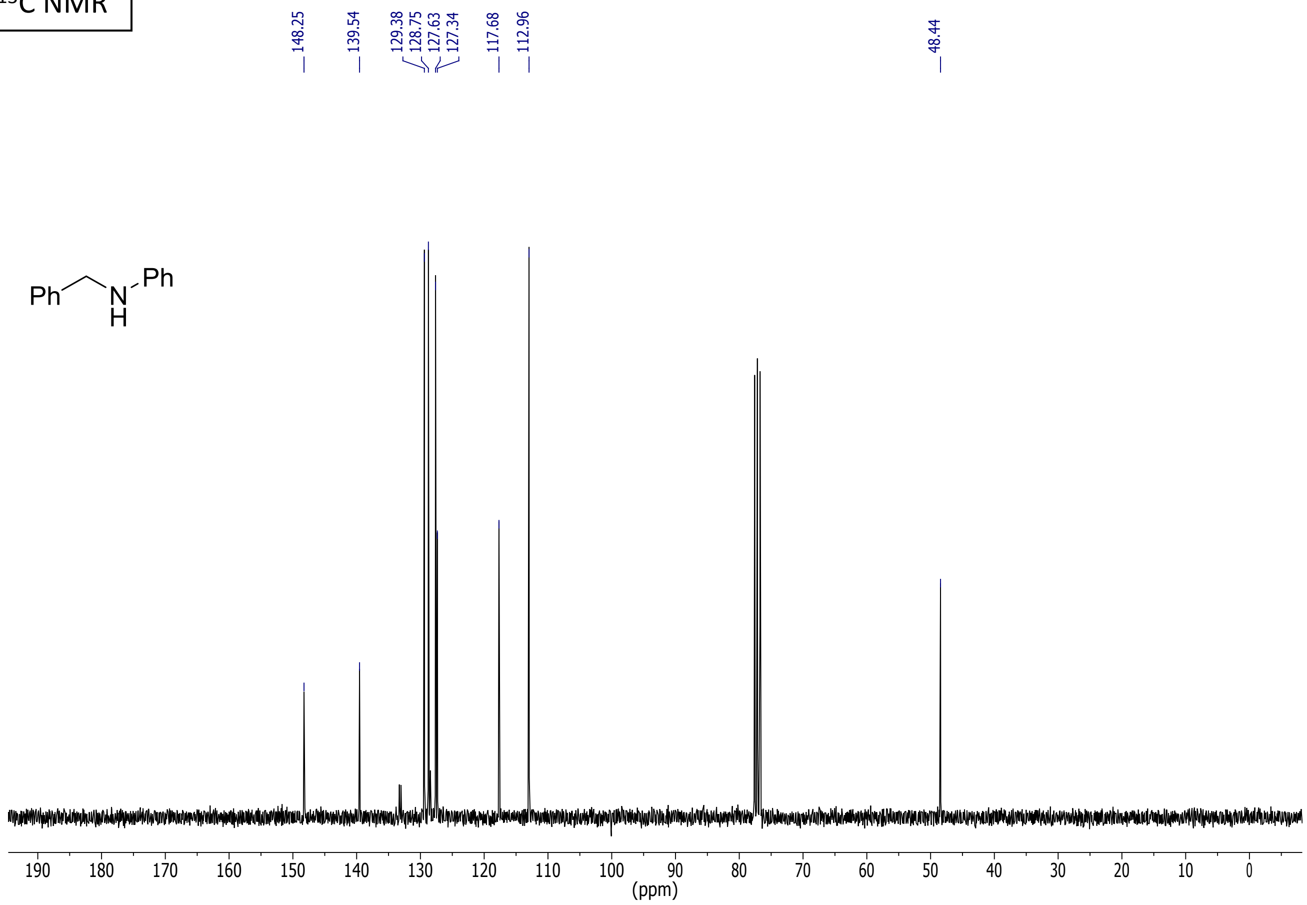

^{1}H NMR

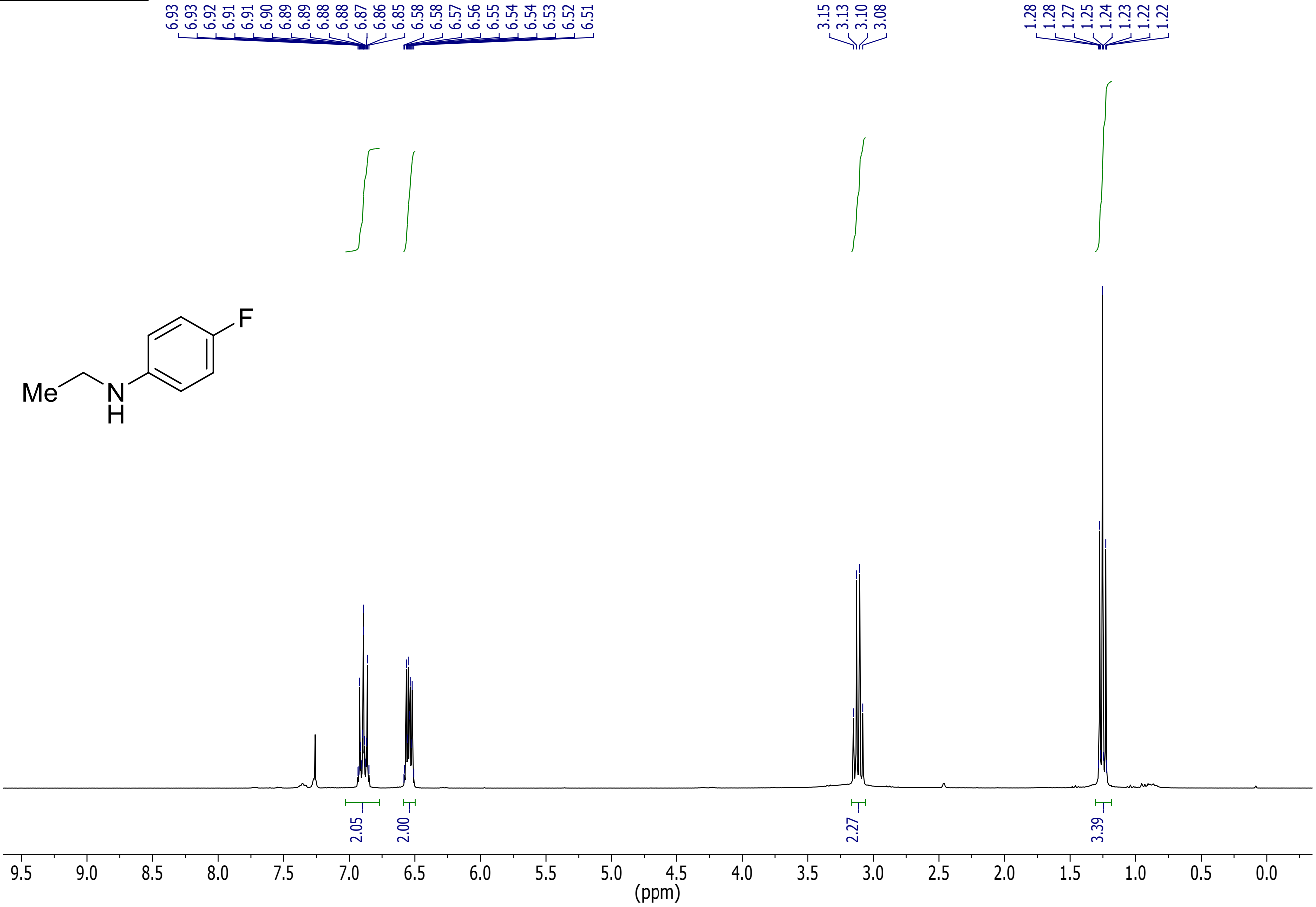

^{13}C NMR

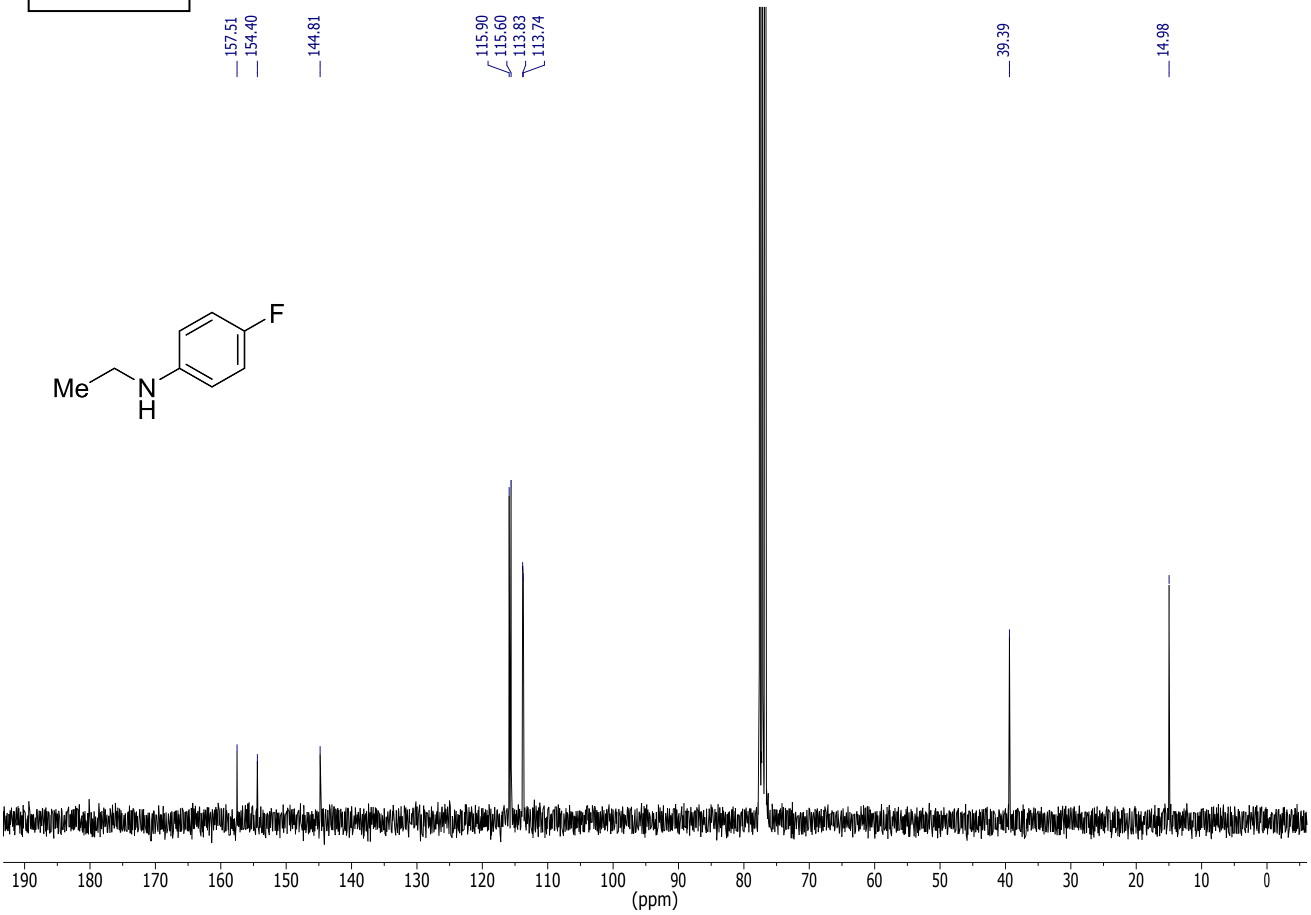

^{19}F NMR

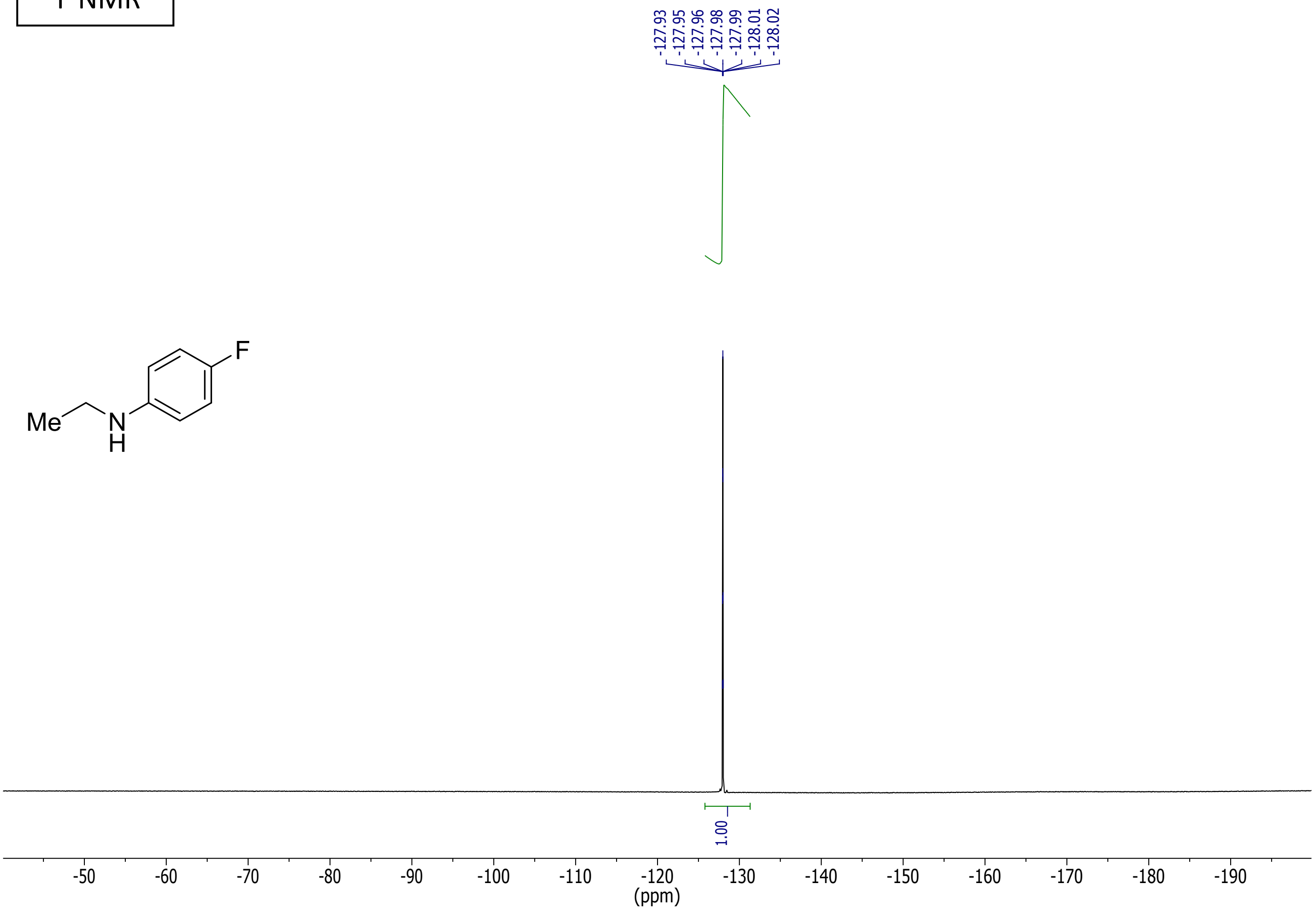

-127.93
-127.95
-127.96
-127.98
-127.99
-128.01
-128.02
F
Me
N
H
1.00
-50
-60
-70
-80
-90
-100
-110
-120
-130
-140
-150
-160
-170
-180
-190
(ppm)

^{1}H NMR

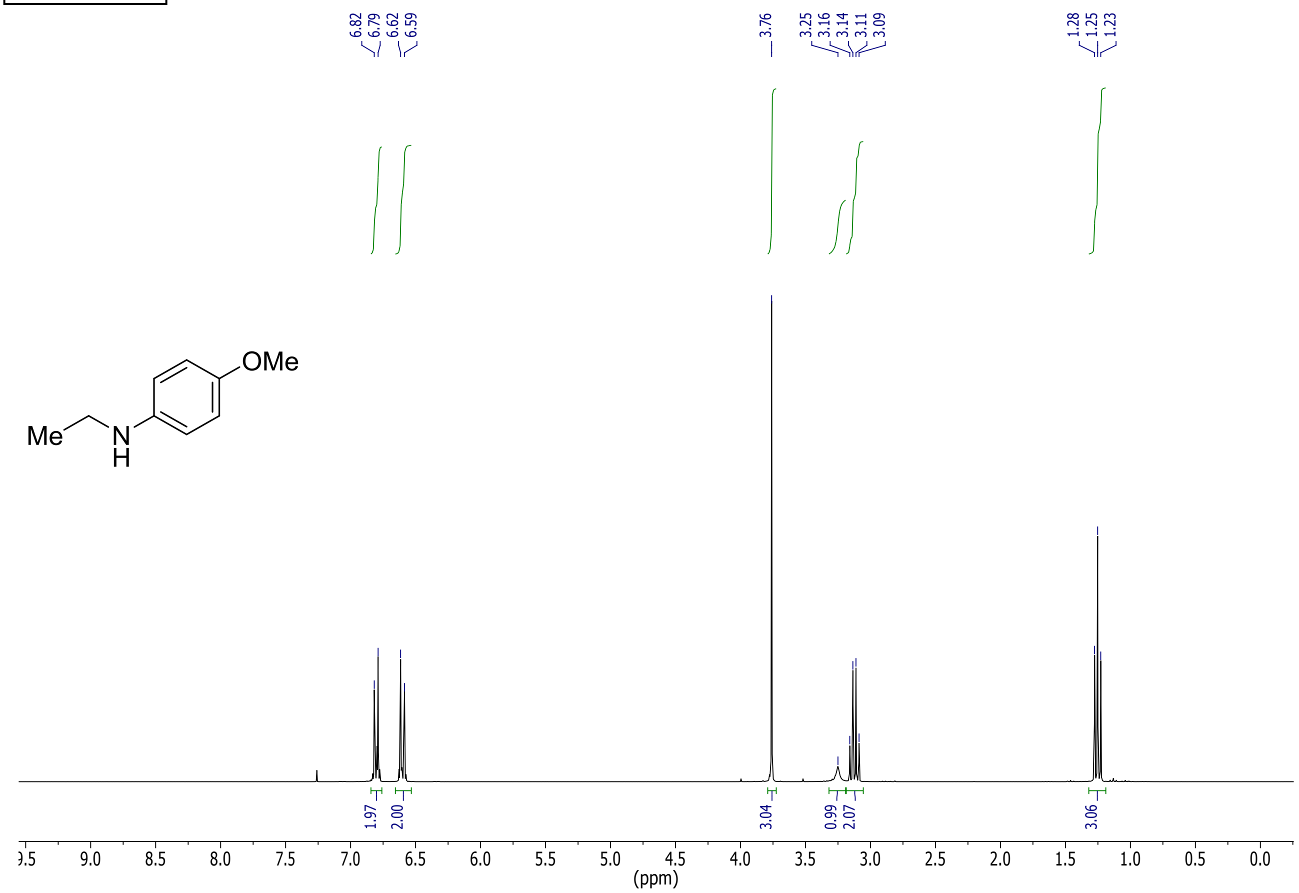

^{13}C NMR

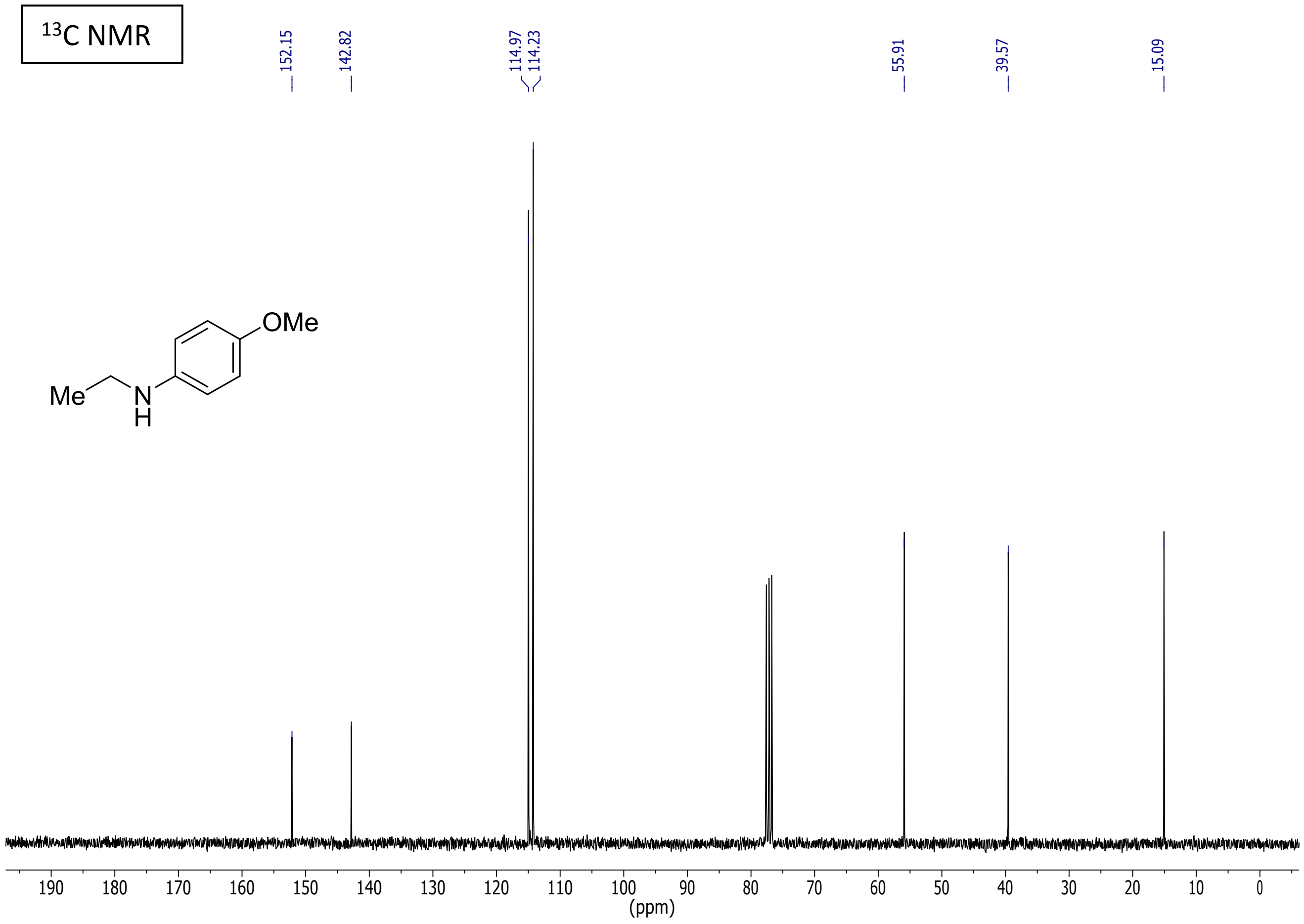

^{1}H NMR

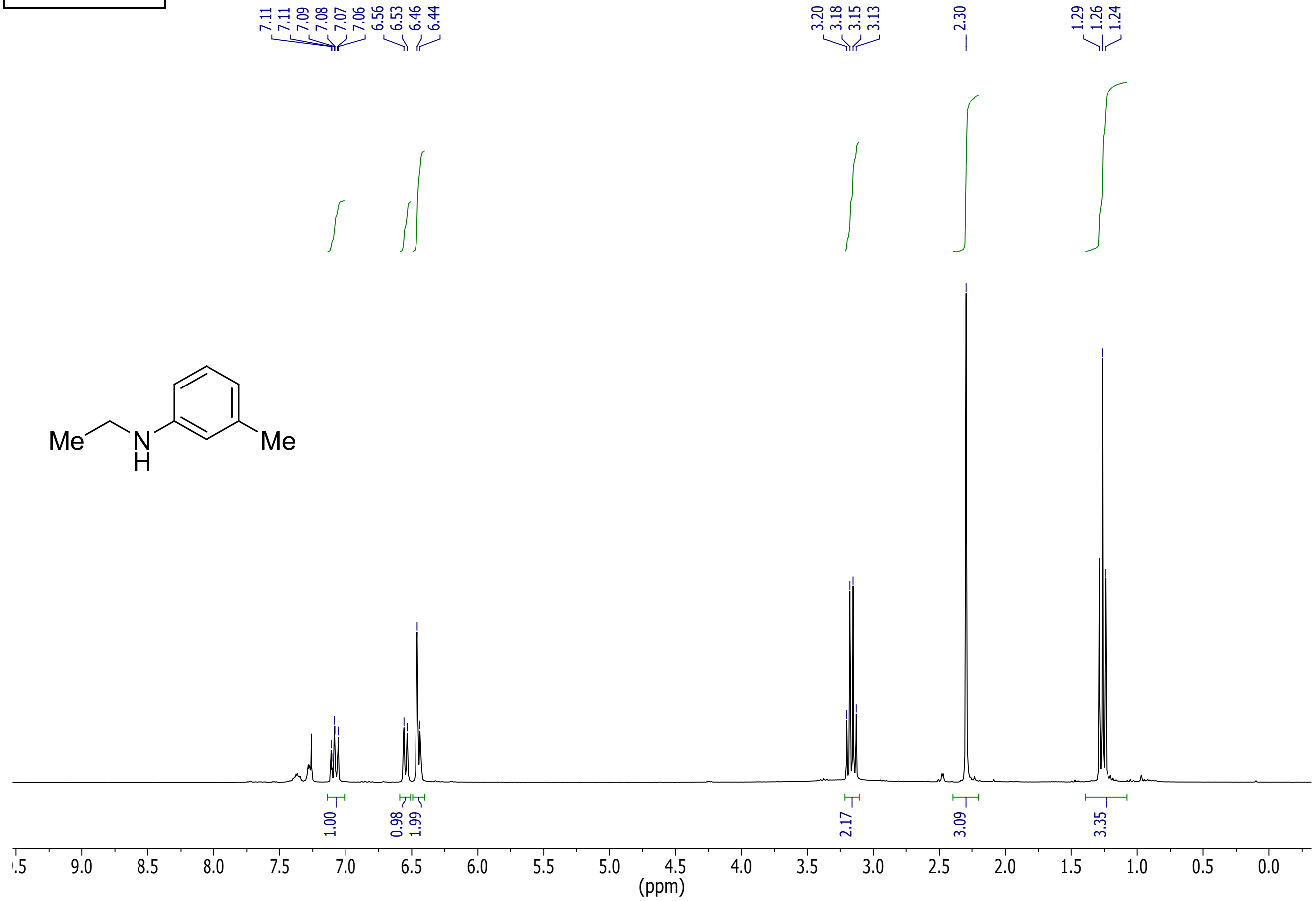

^{13}C NMR

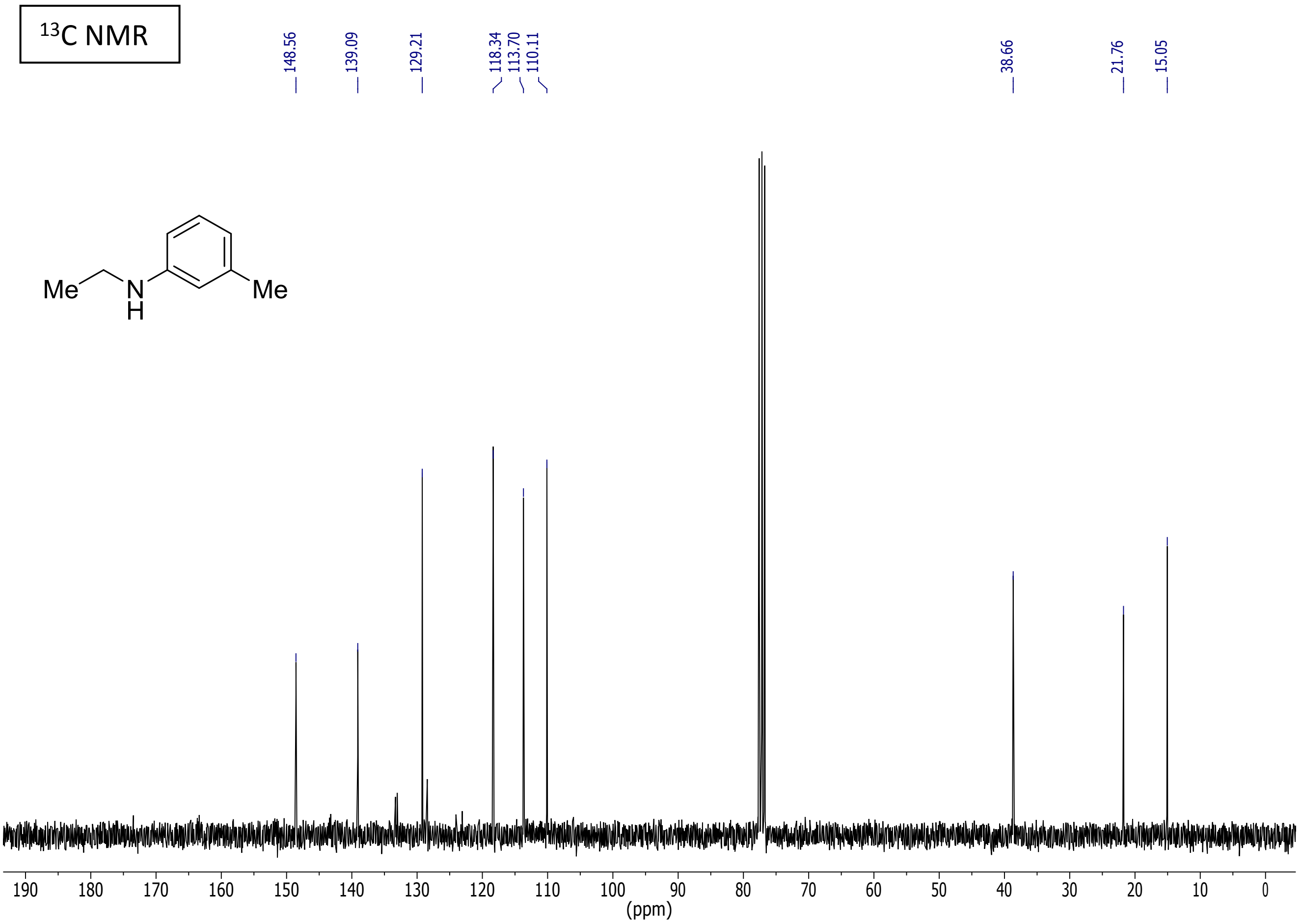

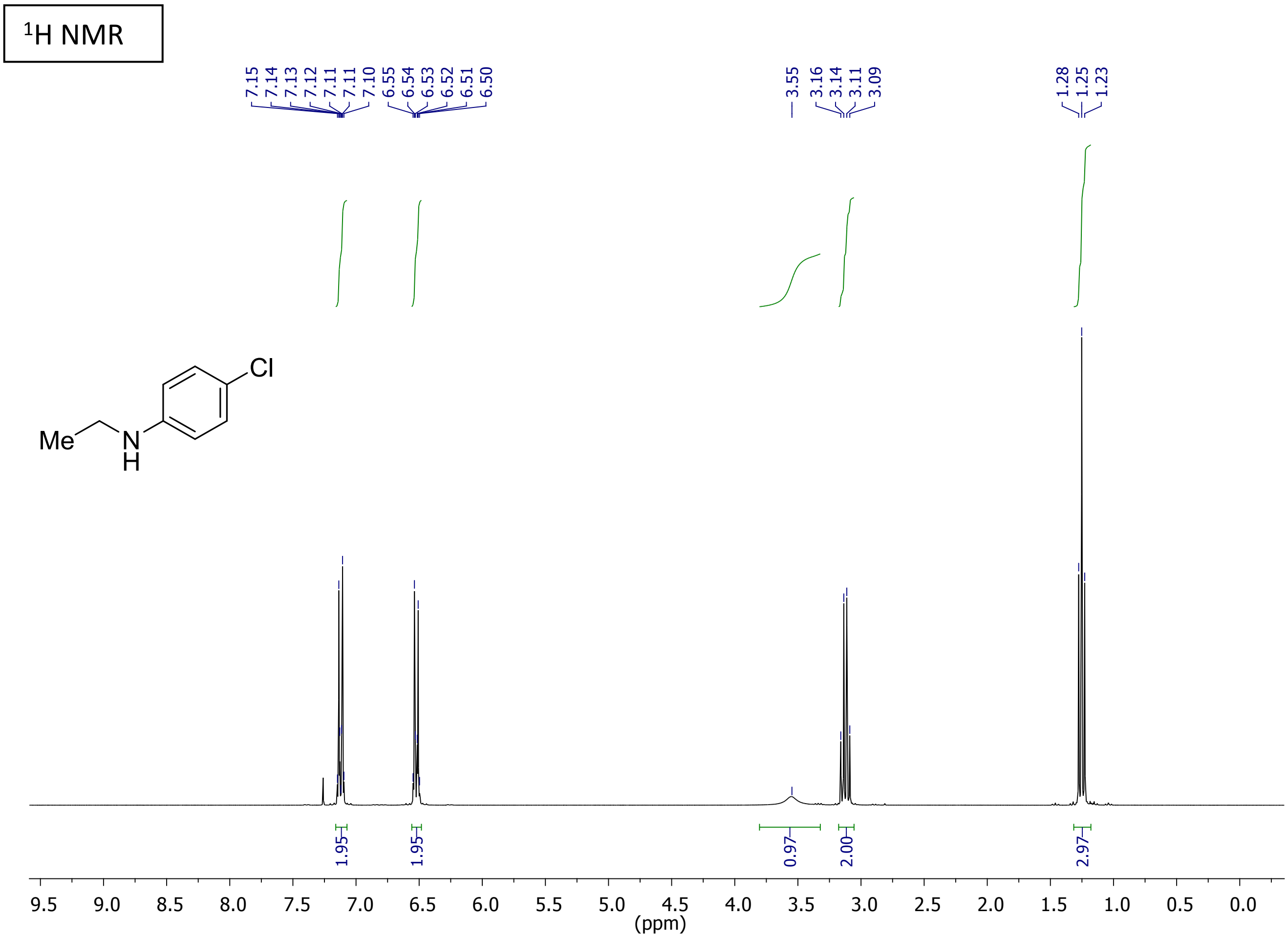

1H NMR
7.15
7.14
7.13
7.12
7.11
7.11
7.10
6.55
6.54
6.53
6.52
6.51
6.50
3.55
3.16
3.14
3.11
3.09
1.28
1.25
1.23
Cl
Me
N
H
1.95
1.95
0.97
2.00
2.97
9.5 9.0 8.5 8.0 7.5 7.0 6.5 6.0 5.5 5.0 4.5 4.0 3.5 3.0 2.5 2.0 1.5 1.0 0.5 0.0
(ppm)

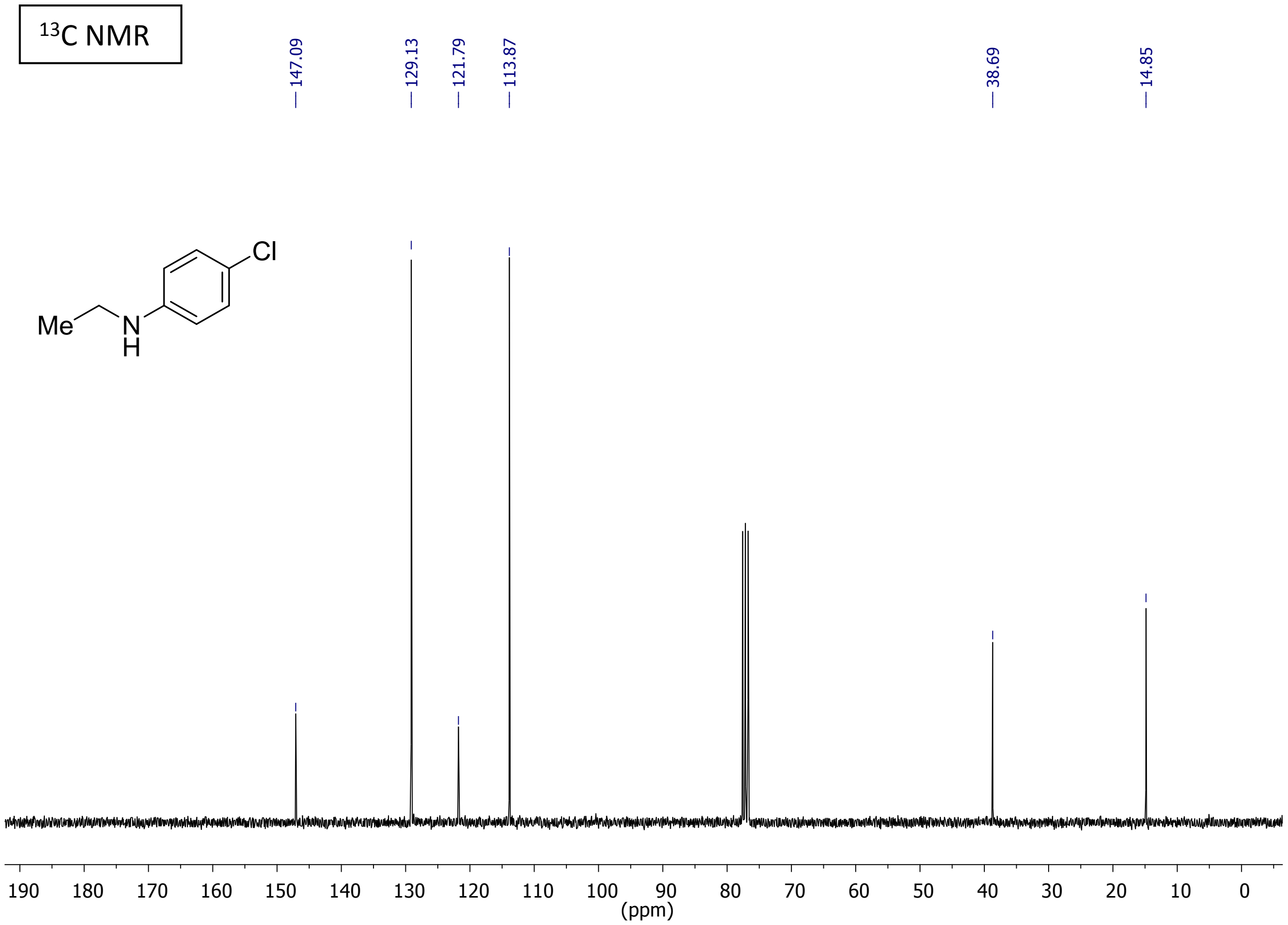

13C NMR
147.09
129.13
121.79
113.87
38.69
14.85
Cl
Me
N
H
190 180 170 160 150 140 130 120 110 100 90 80 70 60 50 40 30 20 10 0
(ppm)

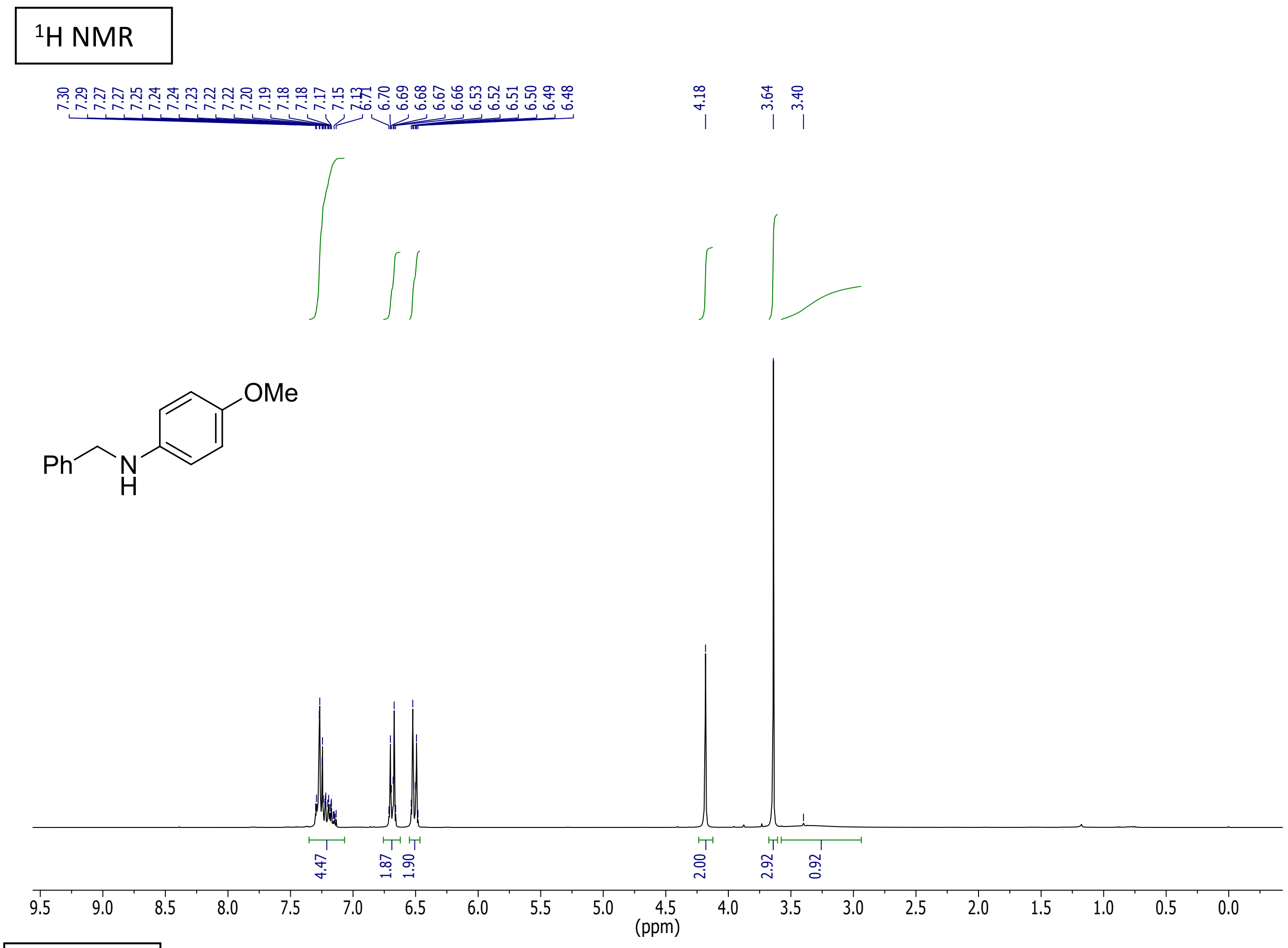

1H NMR
7.30 7.29 7.27 7.27 7.25 7.24 7.24 7.23 7.22 7.22 7.20 7.19 7.18 7.18 7.17 7.15 7.13 6.71 6.70 6.69 6.68 6.67 6.66 6.53 6.52 6.51 6.50 6.49 6.48
4.18
3.64
3.40
OMe
Ph
N
H
4.47
1.87
1.90
2.00
2.92
0.92
9.5 9.0 8.5 8.0 7.5 7.0 6.5 6.0 5.5 5.0 4.5 4.0 3.5 3.0 2.5 2.0 1.5 1.0 0.5 0.0
(ppm)

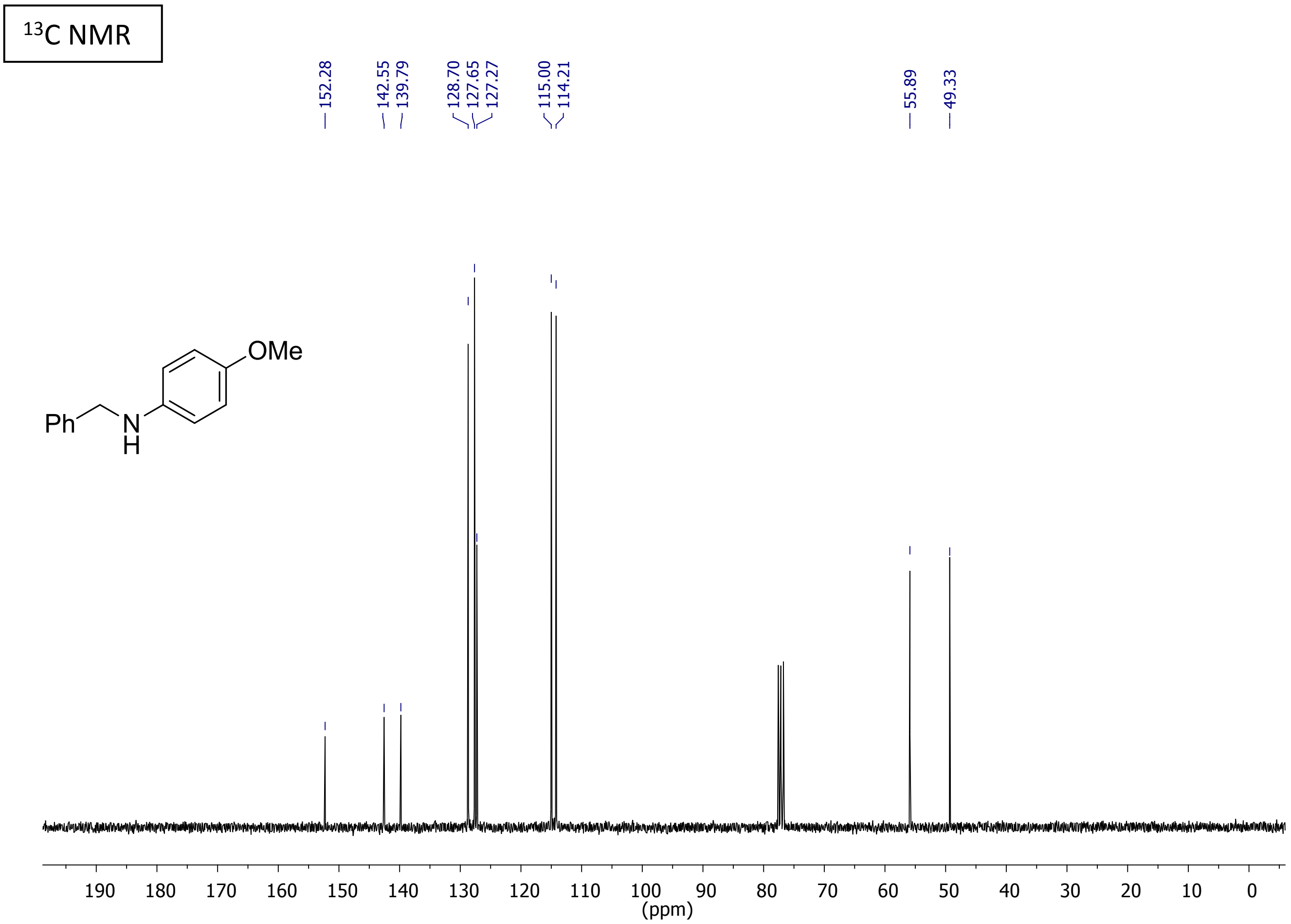

13C NMR
152.28
142.55
139.79
128.70
127.65
127.27
115.00
114.21
55.89
49.33
OMe
Ph
N
H
190 180 170 160 150 140 130 120 110 100 90 80 70 60 50 40 30 20 10 0
(ppm)

^{1}H NMR

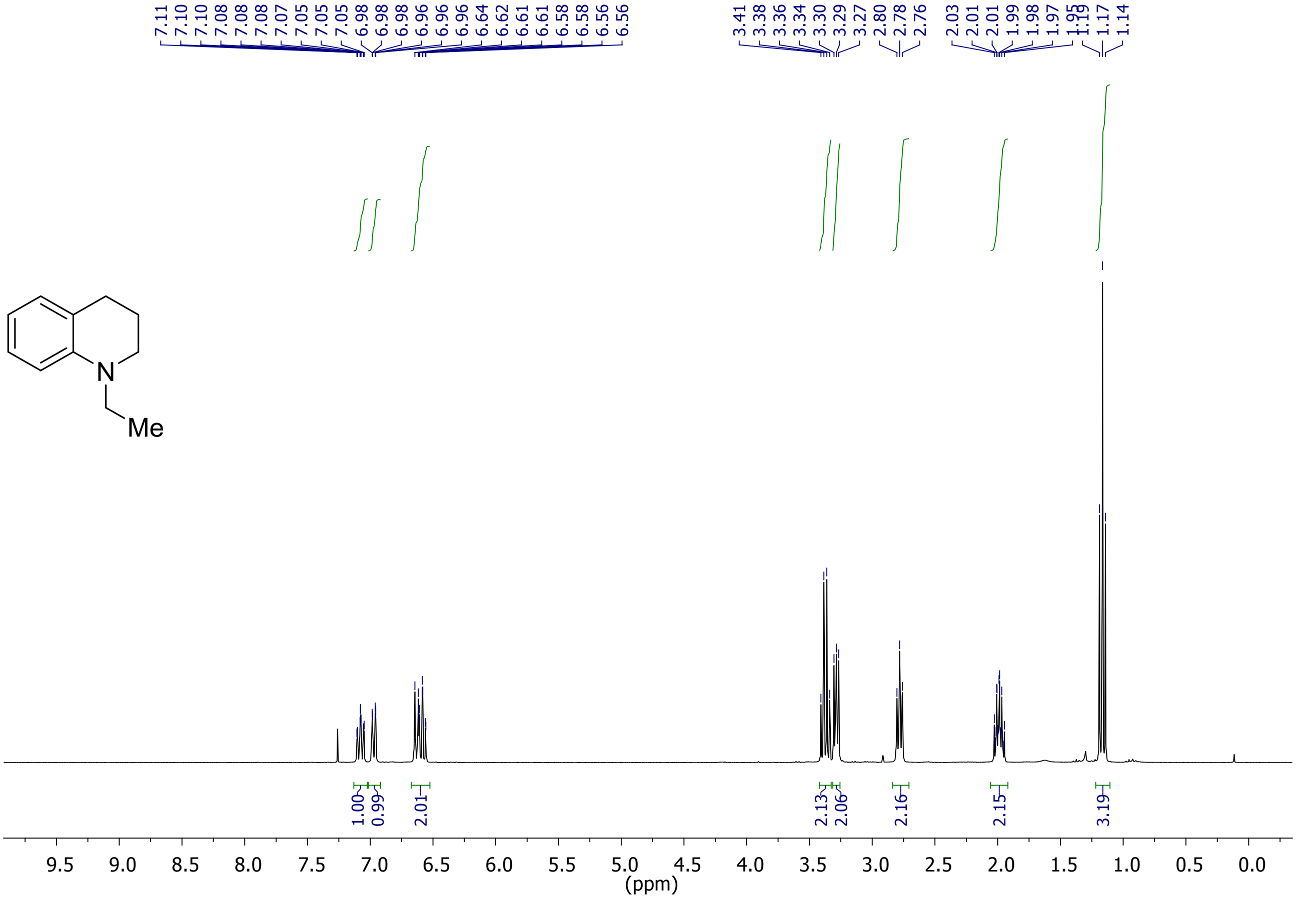

^{13}C NMR

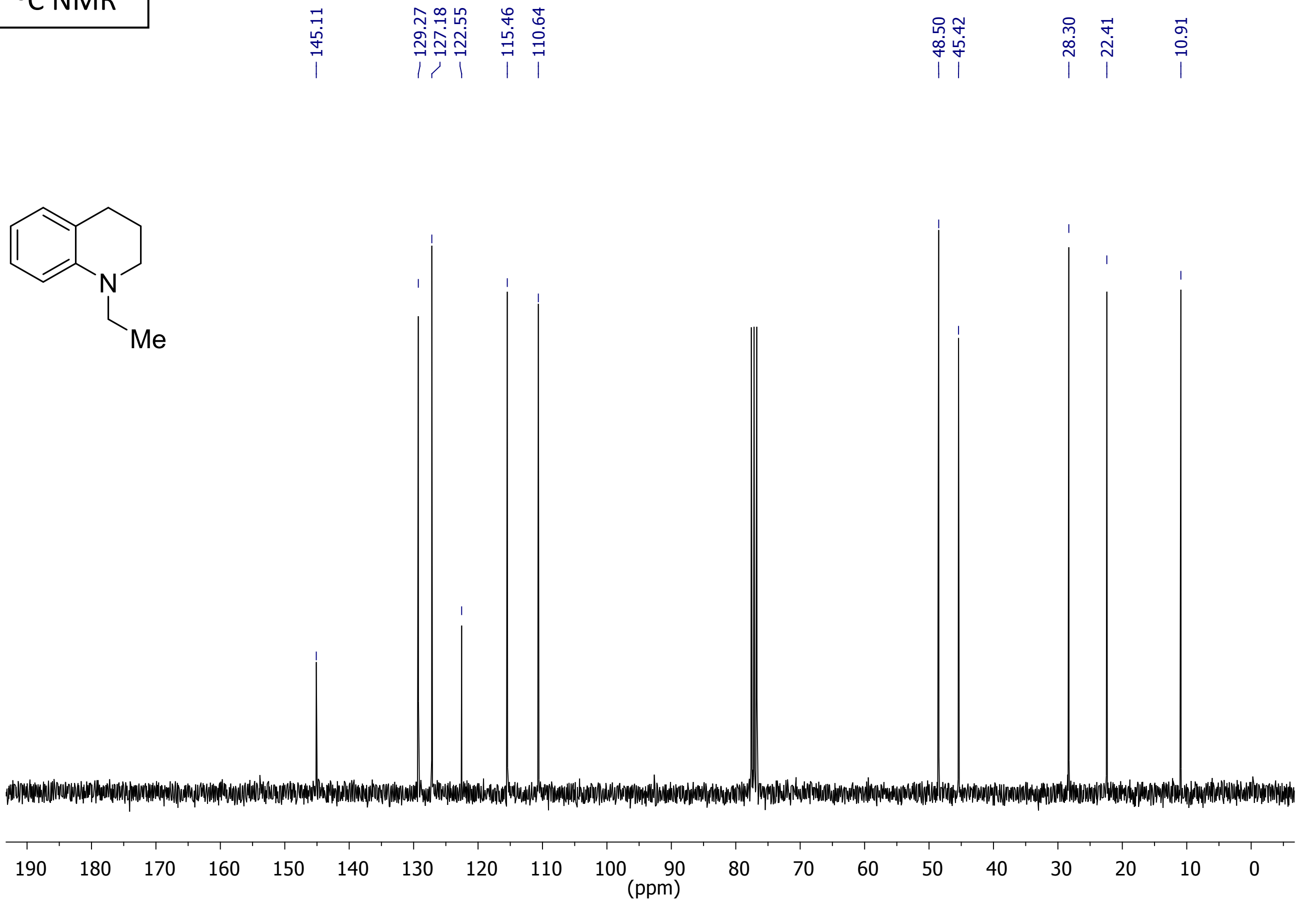

^{1}H NMR

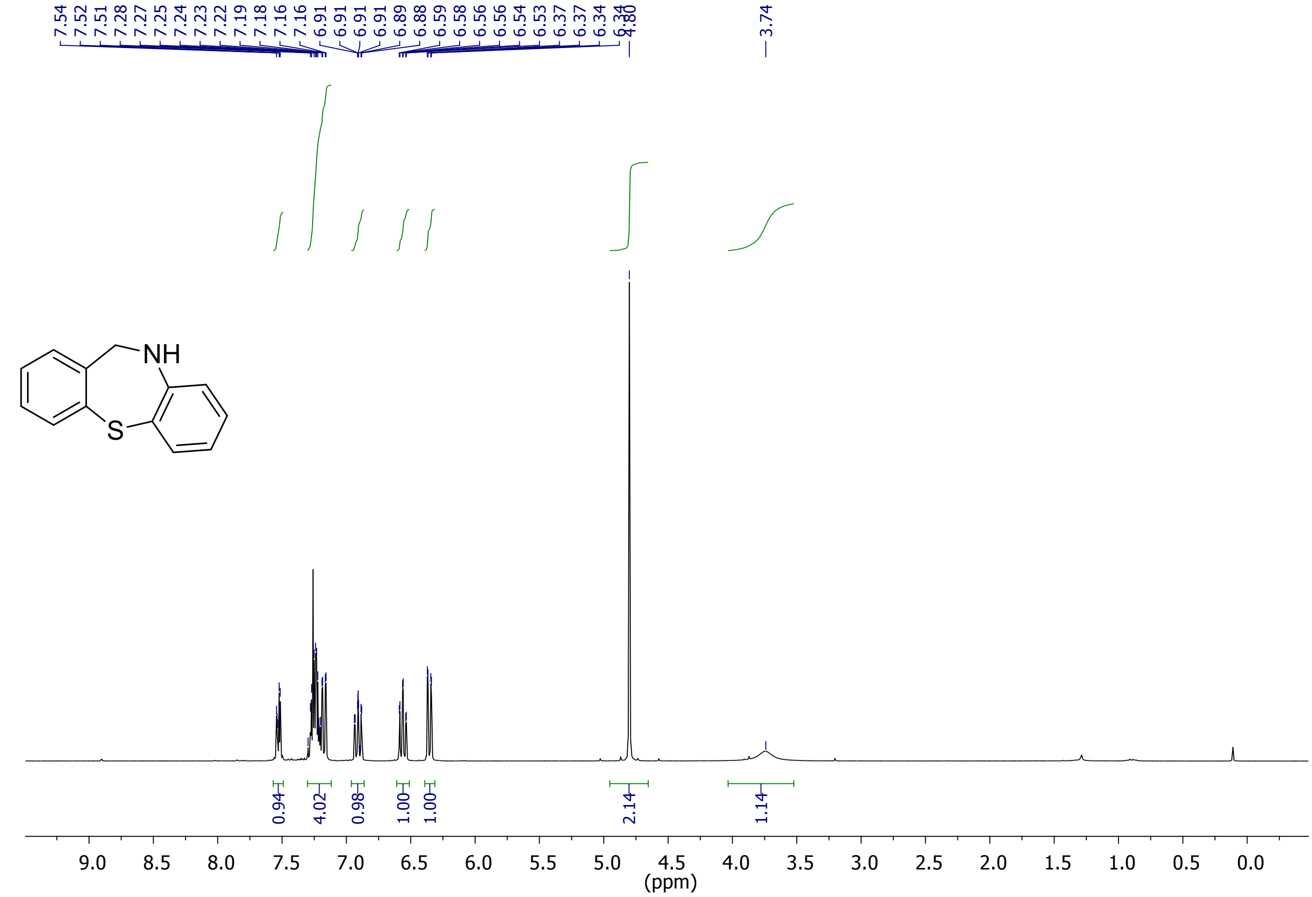

^{13}C NMR

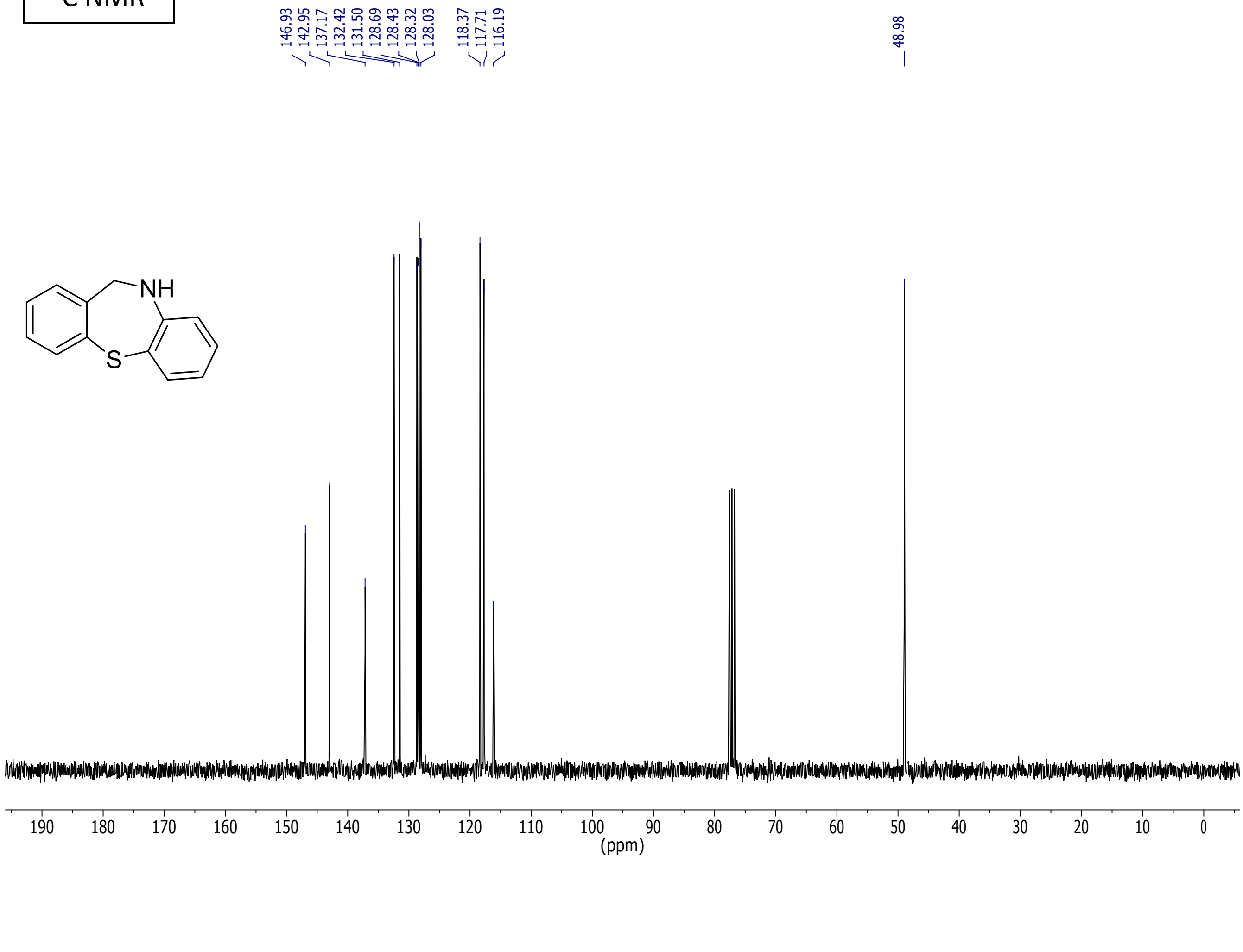

^{1}H NMR

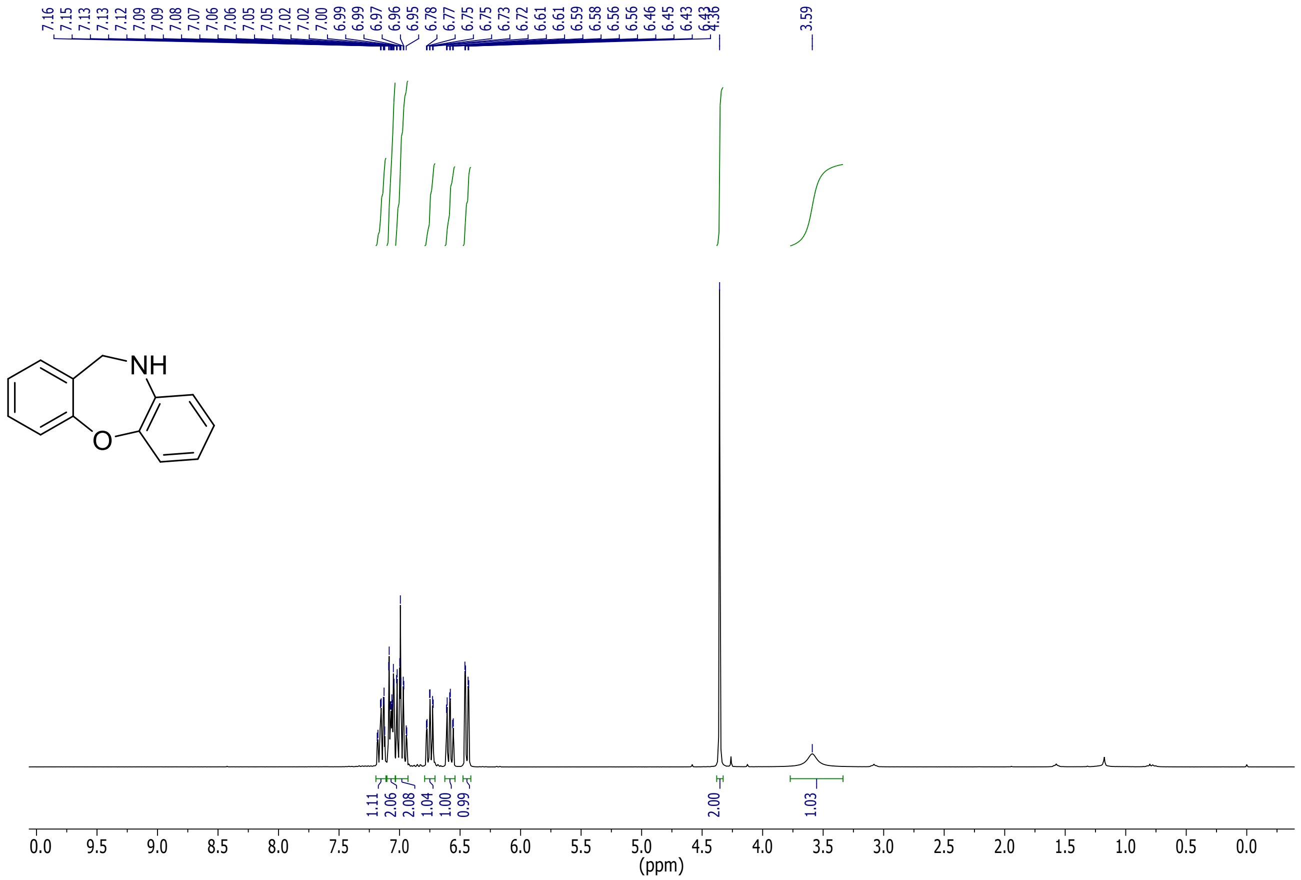

^{13}C NMR

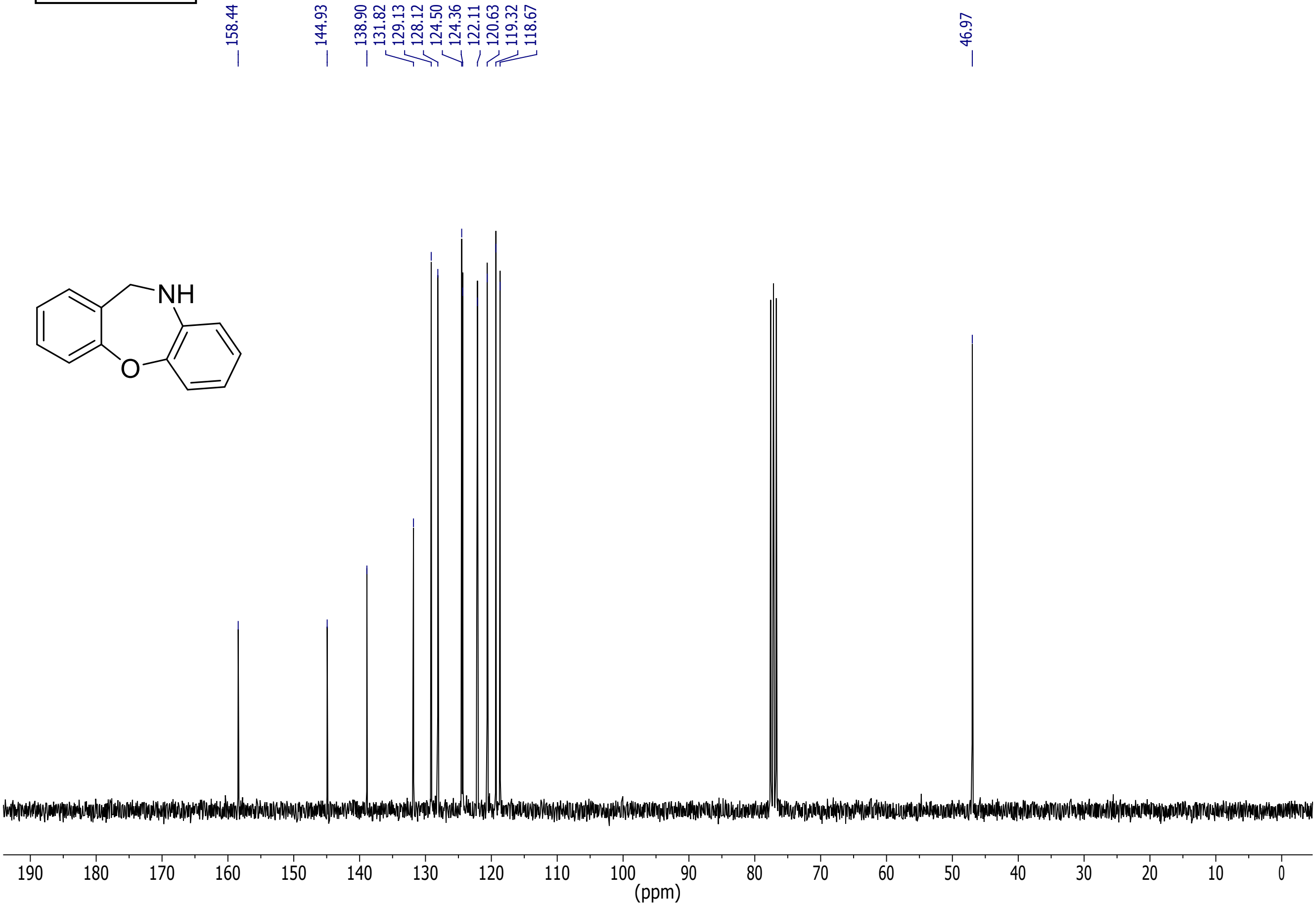

^1H NMR

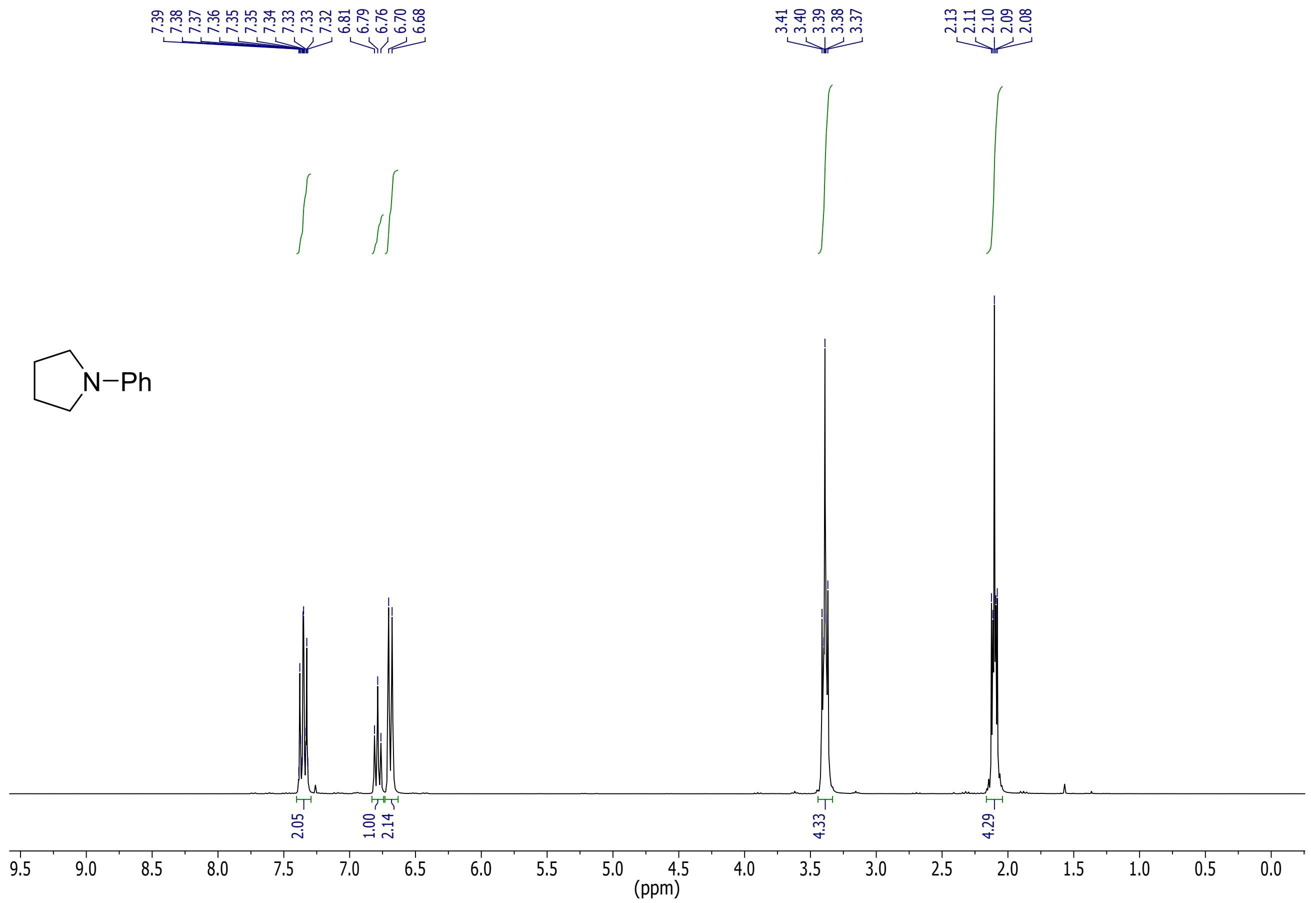

^{13}C NMR

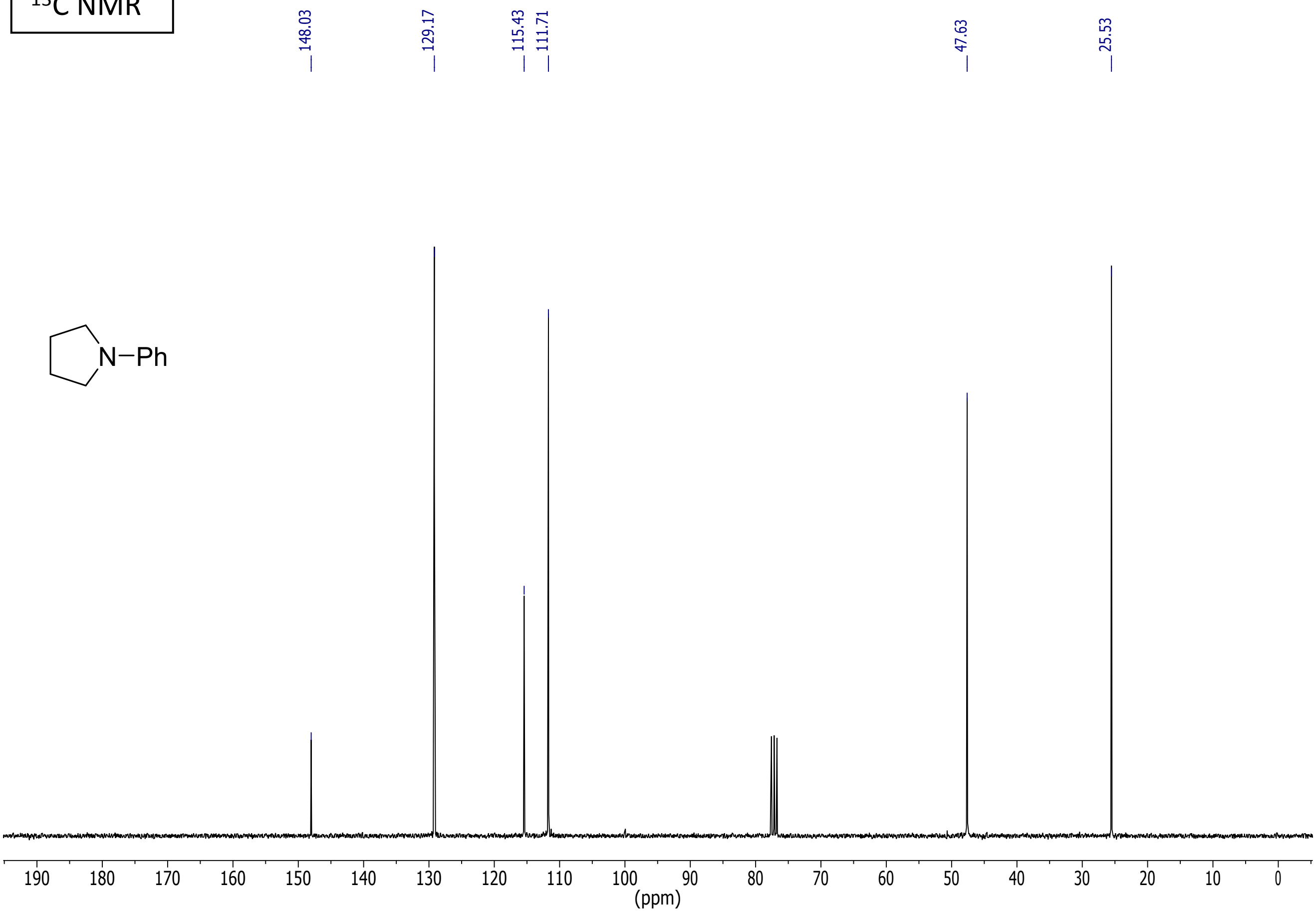

Supplement: Supplementary file 1 [file SC-007-C5SC04671H-s001.pdf]
